# Prefrontal and Subcortical c-Fos Mapping of Reward Responses across Competitive and Social Contexts

**DOI:** 10.1523/ENEURO.0158-25.2025

**Published:** 2025-11-06

**Authors:** Caroline De Paula Cunha Almeida, Meghan Cum, Elizabeth Illescas-Huerta, Amelia Chambers, Charles Ye, Aarna Shah, Ayush Jain, Albert Li, Nancy Padilla-Coreano

**Affiliations:** Department of Neuroscience, College of Medicine, University of Florida, Gainesville, Florida 32610

**Keywords:** c-Fos, dominance, prefrontal, reward, social context

## Abstract

Social animals compete for rewards to survive, yet the neural circuits underlying reward-based social competition remain unclear. The medial prefrontal cortex (mPFC) plays a key role in reward processing and social dominance, but whether its subregions contribute differently to competitions for reward remains unknown. Using c-Fos mapping in male CD1 mice, we examined reward-induced neural activation in mPFC subregions and key interconnected subcortical areas across social and nonsocial reward contexts. Noncompetitive social contexts produced global c-Fos activation relative to competitive contexts. Cross-regional correlation analyses revealed that receiving rewards in isolation involved widespread network coordination, while social contexts exhibited distinct, sparse correlation patterns. Surprisingly, social rank effects on neural activity were most pronounced during isolated reward experiences rather than during competition, with dominant mice showing increased anterior cingulate, basolateral amygdala, and hippocampal activation when alone. Different dominance ranks (reward-based, territorial, and agonistic) correlated with distinct neural activity patterns across contexts. Overall, our results show that social context fundamentally reorganizes prefrontal–subcortical networks during reward processing in a social rank-dependent manner. These results provide new insights into how social rank shapes the neural basis of reward processing across different social contexts.

## Significance Statement

Social competition for rewards is fundamental for the survival of social species, yet the neural circuits supporting it remain poorly understood. We use mice to show that medial prefrontal cortex subregions and interconnected subcortical areas exhibit distinct activation and correlations depending on the social context in which the rewards are obtained. Social rank in mice influenced neural activation patterns, with subordinate mice showing increased activation in subcortical regions during exposure to noncompetitive rewards. These findings highlight that reward processing in key subcortical regions within and connected to the mPFC are shaped by social context and hierarchy, providing insight into the neural mechanisms underlying social competition.

## Introduction

In both humans and animals, competition for limited resources is a fundamental aspect of social behavior, with rewards serving as potent drivers of decisions and interactions. Competition shapes social hierarchies that are critical for survival across species, yet the underlying neural mechanisms of competitive behavior in the context of rewards remain unknown.

The medial prefrontal cortex (mPFC) has emerged as a critical hub for regulating reward competition behaviors, particularly in the context of social hierarchies (Wang et al., 2011; Li et al., 2022; Padilla-Coreano et al., 2022). Within the mPFC, distinct subregions contribute differently to reward processing (Burgos-Robles et al., 2013), and they may also contribute differently to social competition. Optogenetic evidence demonstrates that stimulating the prelimbic (PL), but not infralimbic (IL) nor the anterior cingulate cortex (ACC), increases winning in social dominance tests that do not include a reward, such as the tube test (Zhou et al., 2017). However, the ACC is implicated when animals compete for food rewards (Li et al., 2022), suggesting that the presence of reward modulates which cortical circuits are recruited during social competitions. Furthermore, the PL and IL subdivisions of the mPFC play opposing roles in regulating reward memories (Peters et al., 2009), suggesting that these subregions may differentially regulate competitions for reward.

Despite recent advances in understanding the role of the mPFC in reward competition, critical questions remain about which subcortical regions interact with the mPFC at the circuit and network levels during these competitive behaviors. While recent work has shown that manipulating PL neurons projecting to the lateral hypothalamus (LH) increases winning in competitions for liquid rewards (Padilla-Coreano et al., 2022), the broader subcortical network engaged during reward competition remains largely unexplored. To address this gap, we examined candidate regions previously implicated in social hierarchy through nonreward paradigms, like the tube test: the basolateral amygdala (BLA), which forms important connections with the mPFC for sociability (Felix-Ortiz and Tye, 2014) and mPFC to BLA activity influences tube test winning (Xin et al., 2024); the mediodorsal thalamus (MD), whose inputs to the mPFC undergo long-lasting plasticity in response to social competition outcomes (Zhou et al., 2017); the nucleus accumbens (NAc), known for reward learning and reward-seeking behavior (Carelli, 2002; Carlezon and Thomas, 2009) but has been recently linked to competitive outcomes in the tube test (Shan et al., 2022); and the ventral CA1 of the hippocampus (vCA1), necessary for both social and reward memory expression (Riaz et al., 2017; Phillips et al., 2019). While these regions have established roles in nonreward social competitions like the tube test, their specific contributions to reward-based social competition remain an open question. Investigating how these regions relate to reward-based competition is critical for our understanding of the mechanisms governing social hierarchies and may have translational relevance for diseases that impact social functioning in humans.

We hypothesized that mPFC subregions and these key subcortical regions would show different activity and neural patterns when mice compete for rewards. To test our hypothesis, we characterized network activity across mPFC subregions and associated subcortical regions involved during reward-based competition. We used c-Fos mapping to quantify neural activity levels across these regions during competition for rewards. Next, we determined whether network differences during reward competition were specifically driven by social context rather than competition itself by comparing c-Fos activation patterns between mice experiencing rewards in competitive versus noncompetitive social contexts. Previous findings show that dominant and subordinate mice exhibit distinct mPFC population dynamics when exposed to cued rewards, suggesting that social rank may impact neural responses to reward even outside of a social context (Padilla-Coreano et al., 2022; Patel et al., 2024). We build upon these findings by examining how social rank correlates with neural activation across both social and nonsocial reward contexts.

Here, we demonstrate that social context profoundly shapes neural activity patterns across mPFC subregions and subcortical circuits during reward processing. We found that noncompetitive social reward contexts globally increased neural activation compared with competitive or isolated contexts, while cross-regional functional connectivity patterns were distinctly reorganized in social versus nonsocial settings. Finally, correlations between social dominance rank and neural activity were context-dependent. These findings reveal that social hierarchy influences reward processing through context-specific modulation of prefrontal–subcortical networks.

## Materials and Methods

### Animals

All experimental protocols were approved by University of Florida Institutional Animal Care and Use Committee. Male CD1 mice were received from Charles River Laboratories. All experimental animals were housed in a 12 h reverse light cycle (9:00 A.M. to 9:00 P.M.), and all behavioral experiments were conducted during the dark phase. CD1 male mice (*n* = 28) of 3–4 months old were group-housed in cages of four. Mice were food-restricted to 85–90% of their baseline body weight throughout the duration of the experiment. Water was available *ad libitum*. All mice were handled for 3 d before behavioral testing and identified using unique patterns applied with a nontoxic animal marker (Stoelting), and markings were reapplied as needed.

### Behavioral assays

#### Reward training and competition

Male CD1 mice were trained to associate a tone with a liquid reward during 10 daily sessions. Tone–reward associations were considered learned once mice reliably approached the reward port following tone presentation. Mice were trained for 10 d prior to the competition. For training sessions, mice were individually placed into a Med-PC operant chamber (Med Associates) with an adapted, custom-made, 3D-printed reward port. A 10 s tone played at 70 dB at pseudorandomized intervals served as a cue for the reward. Mice received 15 μl of vanilla Ensure as a reward 4 s after tone onset. Port entries were detected via infrared beam breaks. Latency (defined as first port entry after tone onset) was calculated and assessed during training to confirm that the mice learned to associate the tone with reward delivery. Mice were rotated across Med-PC operant chambers every day during training, and all cage members were trained individually at the same time for 1 h training sessions. All individual mice achieved an average latency to port entry of 10 s or less after tone onset by Day 9 of training. Due to data file loss on Day 10, we were able to reliably use latency data from Day 9 of training for statistical comparisons of learning across animals. All mice had already met learning criteria by this time point. After reward training, competitions began for 2 consecutive days. Mice were subject to three competition sessions per day (six sessions in total) against each cagemate daily with 2 h breaks in-between matches. The order of matches was counterbalanced across competition days. Competition sessions lasted 31 min (19 trials). Video of the training and competition was recorded at 30 fps. Each competition video was scored by blinded trained investigators, and all individual animals received a ranking based on wins, losses, and ties relative to their cagemates. Elo scores were calculated by treating each of the 19 trials within a session as a separate match. Additionally, reward competition videos were analyzed for the number of bites, chases, and fights observed. Agonistic interactions were summed across all sessions. Each mouse was ranked relative to its cagemates based on initiated and received interactions, with each interaction acting as a trial such that the initiator would be marked as the winner and the receiver would be marked as the loser.

#### Urine marking

Mice were placed in a 3D-printed, custom-made, clear plastic enclosure (16.5 × 39.0 cm) with removed bedding and paper flooring in a dimly lit room. The enclosure was split into two sections by a clear perforated wall, allowing visual and olfactory interaction between the mice on either side. Chromatography paper (Whatman cellulose) was positioned beneath the arena and covered by wire mesh to prevent the mice from chewing the paper. The mice remained in the arena for 2 h. Each mouse was tested against a different cagemate across 3 d, and the number of urine markings was hand-counted under UV light for each session. These markings were used to calculate Elo scores to determine territorial-based social ranks.

#### Experimental groups and perfusions

After 10 d of training and 2 d of reward competition, mice were randomly assigned to a context group. Competition context: Dyads of cagemates (*n* = 10 mice) were placed together in the operant chamber and competed against each other for rewards during 31 min sessions for 19 trials. Together context: Dyads of cagemates (*n* = 8 mice) were placed in the operant chamber for 31 min sessions (19 trials) and separated by a mesh divider. The divider enabled visual and olfactory social interaction without direct competition. Each side of the operant chamber had its own reward port that delivered a reward to both mice during the tone as described above. When a tone played within the operant chamber, rewards were dispensed simultaneously to both mice, with each retrieving the same amount of rewards, allowing both animals to experience the same number of trials with simultaneous exposure to tone and reward. Alone context: Mice (*n* = 10 mice) were placed individually in the operant chamber for a 31 min session with 19 cued rewards. Ninety minutes after the midpoint of the final reward exposure in the assigned context (Competition, Together, or Alone), mice were killed via injection with pentobarbital (31.5 mg/ml) and perfused for c-Fos immunohistochemistry and histological analysis.

#### Histology

Mice were anesthetized with pentobarbital (31.5 mg/ml) and perfused transcardially with 0.9% sodium chloride solution followed by 4% paraformaldehyde (PFA) in 1× phosphate-buffered saline (PBS). Extracted brains were stored in 4% PFA overnight and then transferred into a 30% sucrose solution in 1× PBS for at least 24 h. Brains were sectioned at 40 μm using a microtome, and sections were stored at 4°C in 0.05% sodium azide until staining.

#### Immunohistochemistry

Brain sections were incubated for 1 h in a blocking solution containing PBS with 3% normal donkey serum (NDS) and 0.2% Triton X-100. Sections were then incubated overnight at 4°C in rabbit monoclonal recombinant IgG (Synaptic Systems; dilution of 1:5,000 in PBS-T with 3% NDS). The following day, sections were incubated for 2 h at room temperature in goat anti-rabbit IgG (H + L) cross-adsorbed secondary antibody conjugated with Alexa Fluor 555 (Thermo Fisher Scientific; dilution of 1:500 in PBS-T with 3% NDS). Stained sections were mounted with Fluoromount-G with DAPI (Thermo Fisher Scientific). For each brain region, sections were collected at anterior, medial, and posterior coordinates, with slices taken from either brain hemisphere. Within each section, a fixed field of view corresponding to the microscope's 20× magnification window was used to consistently sample a representative area of the region. Regions of interest were manually delineated within this field of view, and the same imaging parameters were applied across animals and brain regions (Extended Data Fig. 2--1). A mean c-Fos positive cell count (c-Fos^+^) was then obtained by averaging the counts from the three sections. In cases where tissue loss occurred, only the available sections were analyzed. Some animals had partial tissue loss during the sectioning, mounting, and staining processes; therefore not all brain regions have the same sample size (*N*_(ACC)_ = 27; *N*_(PL)_ = 28; *N*_(IL)_ = 28; *N*_(NAc)_ = 26; *N*_(MD)_ = 26; *N*_(BLA)_ = 26; *N*_(LH)_ = 26; *N*_(vCA1)_ = 21).

**Figure 1.**
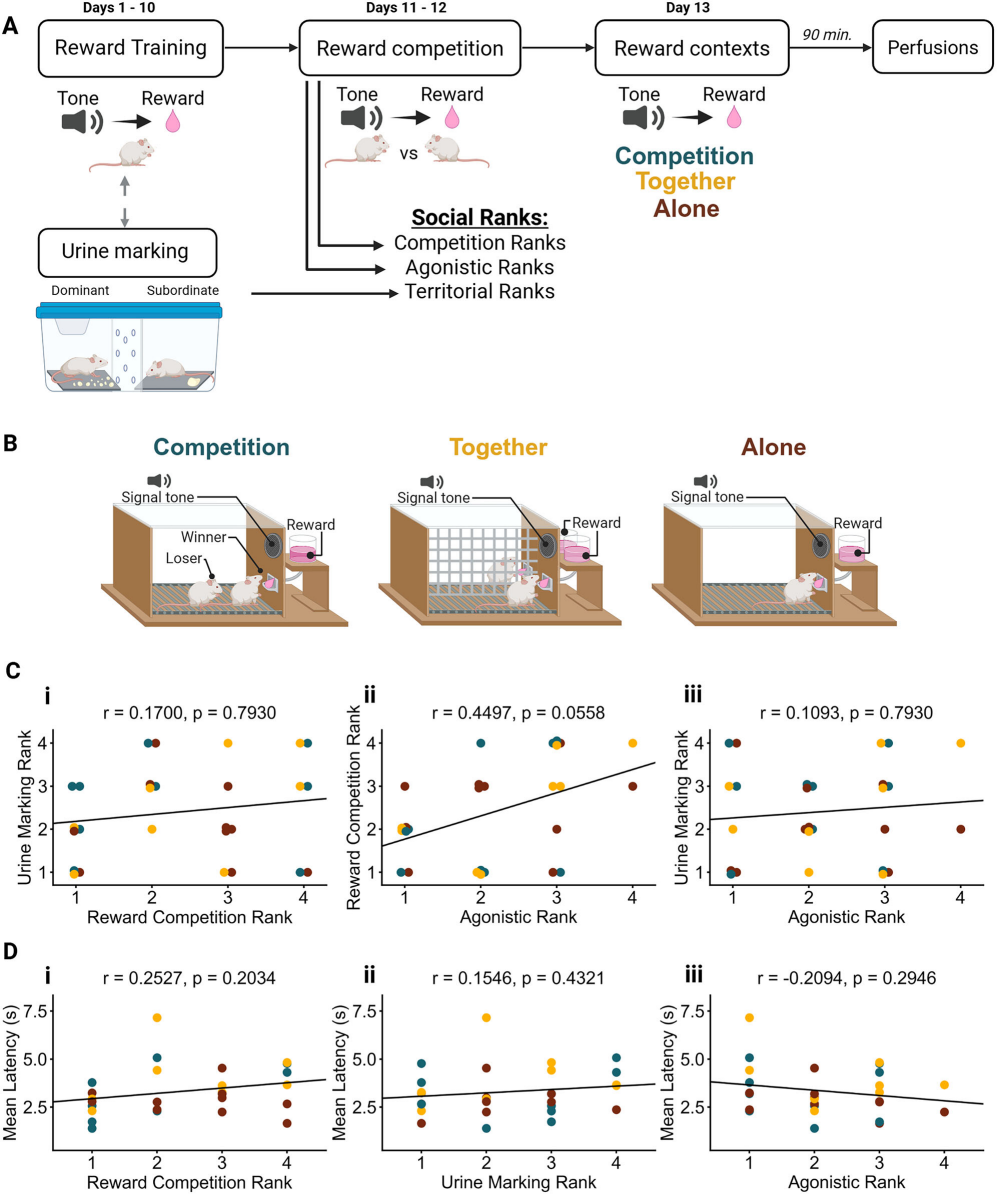
Behavioral procedure across Competitive, Together, and Alone contexts. ***A***, Behavioral timeline. Mice competed against each of their cagemates in a “round-robin” style for urine marking and reward competition for 2 d prior to exposure to reward contexts and perfusion. ***B***, Behavioral paradigm overview: Competition (left/teal, *n* = 10 mice), Together (middle/yellow, *n* = 8 mice), and Alone (right/burgundy, *n* = 10 mice). ***C***, Correlations between social dominance measures for (***i***) territorial versus reward-based social ranks (*r*_(25)_ = 0.1700; *p* = 0.7930), (***ii***) reward versus agonistic-based social ranks (*r*_(25)_ = 0.4497; *p* = 0.0558), and (***iii***) territorial versus agonistic-based social ranks (*r*_(25)_ = 0.1093; *p* = 0.7930). ***D***, Correlations between average time to access the reward port after tone onset (latency) during the ninth day of reward training (see Materials and Methods) and (***i***) reward-based ranks (*r*_(25)_ = 0.2527; *p* = 0.2034), (***ii***) territorial-based ranks (*r*_(26)_ = 0.1546; *p* = 0.4321), and (***iii***) agonistic-based ranks (*r*_(25)_ = −0.2094; *p* = 0.2946) across subjects. Each dot represents one animal; colors indicate behavioral condition—Competition (teal), Together (yellow), and Alone (burgundy)—and with the line of best fit derived from a linear regression analysis. Pearson's correlation analysis and *p* values were corrected using Bonferroni–Holm adjustment.

**Figure 2.**
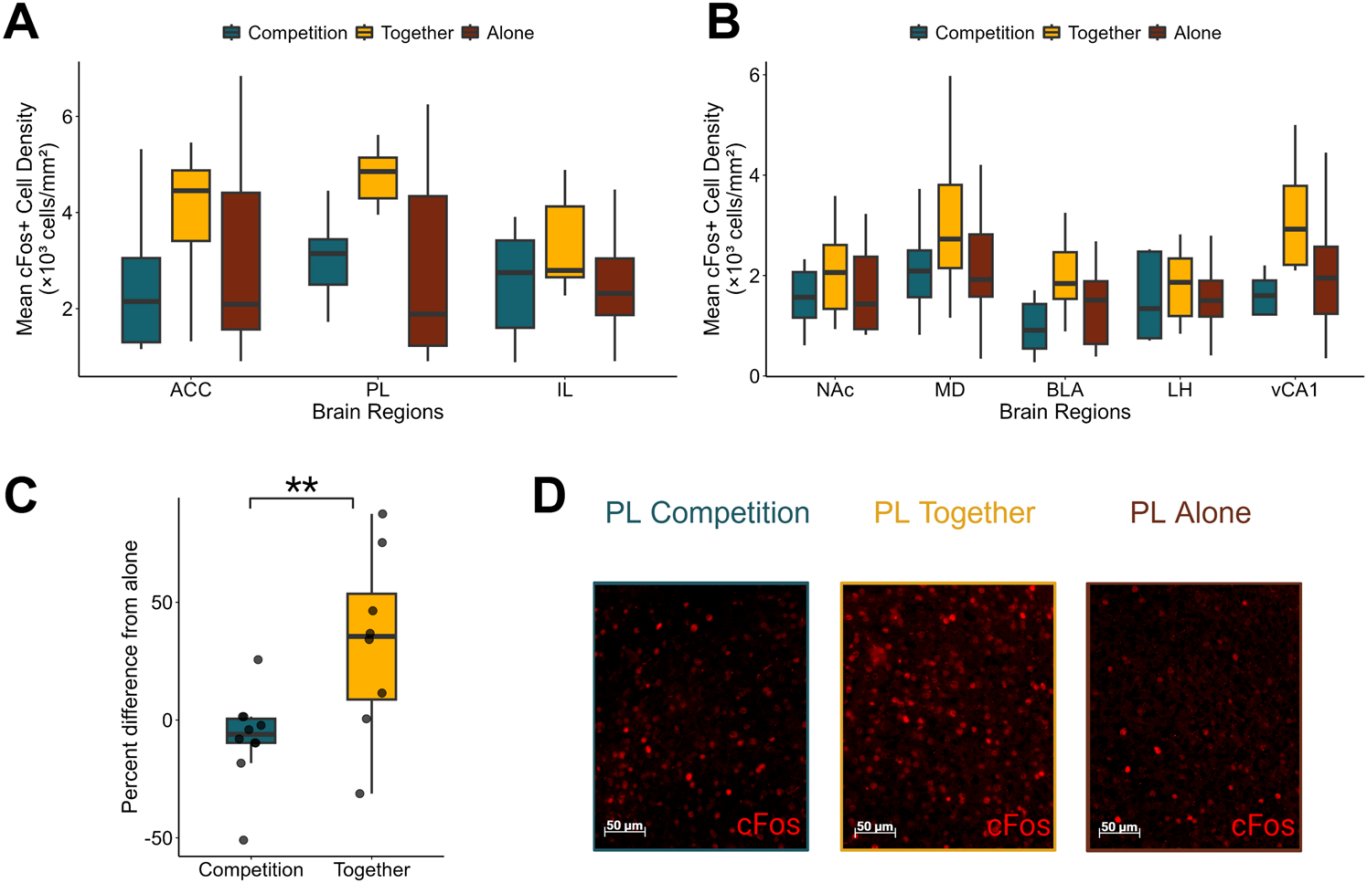
Behavioral context influences c-Fos^+^ cell density and changes from the baseline. ***A***, ***B***, Mean c-Fos^+^ cell density across ACC, PL, IL, NAc, MD, BLA, LH, and vCA1 under three behavioral contexts: Competition (teal), Together (yellow), and Alone (burgundy). Two-way mixed–design ANOVA showed a significant main effect of brain region (*F*_(7, 158.289)_ = 19.5113; *p* = 2 × 10^−16^), but no significant main effect of condition (*F*_(2, 24.607)_ = 2.6438; *p* = 0.0912) and no significant brain region × condition interaction (*F*_(14, 158.279)_ = 1.1756; *p* = 0.2988). ***C***, Global percent difference in c-Fos^+^ cell density normalized to Alone baseline across conditions. Each data point represent the mean difference from the baseline for an individual subject averaged across all brain regions. Boxes show median and interquartile ranges. Two-way mixed–design ANOVA showed a significant main effect of condition (*F*_(1, 16.057)_ = 7.6011; *p* = 0.0140), with no significant main effect of brain region (*F*_(7, 110.176)_ = 1.5974; *p* = 0.1436) nor interaction (*F*_(7, 110.176)_ = 0.7826; *p* = 0.6032). One-sample *t* tests across conditions (*μ* = 0): Competition, *t*_(16.0)_ = −1.019; *p* = 0.3232; Together, *t*_(15.7)_ = 3.398; *p* = 0.0075. Two-sample *t* test on the estimated marginal means of Competition and Together were conducted, *t*_(15.9)_ = −3.207; *p* = 0.0055. Asterisks indicate significance from two-sample *t* test (*p < 0.05; **p < 0.01; ***p < 0.001). ***D***, Example c-Fos images of PL cell count during the Competition, Together, and Alone contexts. See also Extended Data Figures 2-1–2-3.

10.1523/ENEURO.0158-25.2025.f2-1 Figure 2-1 **Example regions of interest for each brain region.** (**A-H**) Average size and shape of the region of interest (ROI) for a given brain area. (**A-G**): 4.4 × 10^4^ µm^2^ square (ACC, PL, IL, NAc, LH, BLA, MD). (**H**) 2.0 × 10^4^ µm^2^ of pyramidal layer of (vCA1). Download Figure 2-1, TIF file.

### Data analysis

#### Reward competition

Each trial was hand-scored by trained observers, and each trial was assigned a winner/loser or labeled as a tie. A tie indicates any trial in which neither mouse maintained a dominant position within the port (i.e., can reach the reward delivery spout) for longer than 1 s after the fourth second of tone onset. An uncontested trial was when one mouse was not at the port during the tone. Port entries were used as a proxy for reward-seeking behavior, quantified by video scoring and validated with MEDPC data from infrared beam breaks at the reward port in the Alone context. Consecutive entries occurring within 0.5 s were counted as a single entry. Latency to reward port was calculated for each condition. The trials scored included those from the two reward competition days to calculate reward-based Elo scores. Competition context mice were also scored for the competition on perfusion day, but this was only used for number of rewards and c-Fos expression correlation. Elo scores were calculated for each mouse using the number of wins, losses, and ties obtained from hand-scored analyses of the two competition days only, allowing the assessment of relative ranks within the group (Cum et al., 2024). This means that for Competition context subjects, competition outcomes on the day of perfusions were not included in Elo score calculations.

#### Urine marking

Trained counters manually recorded urine markings under UV lights. A tie indicates a match with less than a 20% difference or fewer than five-spot difference between the two mice. Each cage pair underwent two trials, with each mouse being tested once per day.

#### Elo score

The Elo score was calculated using custom Python scripts. The Elo ranking system accounts for ties and temporal shifts in rank (Elo, 1978; Albers and De Vries, 2001). This is important as the reward competition had many ties that would otherwise not be counted toward rank.

The Elo score equation is as follows:

$$E_{A}=\;\frac{1}{1+10^{\frac{R_{B}-R_{A}}{400}}},$$

$$R_{A}^{\prime }=\;R_{A}+K(S_{A}-\;E_{A}).$$

Subscripts *A* and *B* represent the two opponents, and *E_A_* is the expected probability that *A* will win. *R_A_* and *R_B_* are the current ratings of the two opponents. *R'_A_* will be the updated rating of *A* given the outcome. *S_A_* will equal 1 if *A* wins, 0 if *A* loses, and 0.5 if the match is a tie. *K* and 400 are constants that control the sensitivity of rating changes. Given that the reward competitions and urine marking assays are done in a randomized round-robin manner, as to not give extra weight to early contests, *K* = 20 was used (Albers and De Vries, 2001). All mice were assigned an Elo score of 1,000 at the beginning of their trials. Elo scores were calculated separately for each behavioral assay. Each individual mouse received an Elo score based on the results of their robin rounds reward competition trials against every cagemate and another Elo score based on their urine marking trials against their cagemates.

#### Cagemate ranks

Elo scores of animals housed within the same cage were compared. Each mouse was assigned a rank of 1–4 based on their relative Elo scores, with 1 representing the most dominant, highest-ranked mouse in the cage and 4 being the lowest. These rank scores were used as the final measure of social rank within each cage. We opted to use a discrete rank system (1–4) rather than continuous Elo scores because the mice used in each testing group were selected from different home cages, and not all mice within each home cage were included in the experiment, thus the same ELO rank across cages could indicate different relative ranks. Due to camera failure, several round-robin competition videos were lost, directly impacting the total sample for the calculations of the number of rewards obtained during the Competition context and mean number of c-Fos^+^ cells across brain regions (Extended Data Fig. 2-2). That mouse was excluded from analyses that relate to reward competition social rank since an accurate Elo score could not be calculated.

10.1523/ENEURO.0158-25.2025.f2-2 Figure 2-2 **Rewards won during Competition context.** (**A-H**) Scatterplots for the number of rewards obtained during the competition context and mean number of c-Fos + cells across brain regions. Each dot represents an individual animal, with lines of best-fit derived from a linear regression analysis. P-values were adjusted using a Benjamini-Hochberg False Discovery Rate procedure. Pearson correlation coefficient (r) and associated adjusted p-values are displayed above each plot. Download Figure 2-2, TIF file.

#### Immunoreactivity quantification

Brain sections were imaged at 20× magnification using a KEYENCE BZ-X800 all-in-one fluorescence microscope (Keyence Corporation of America) under high-sensitivity settings. Maximum projection images were generated using the Full Focus option. Images were generated for the ACC (bregma 2.33–1.33 mm), PL (bregma 2.33–1.33 mm), IL (bregma 1.97–1.33 mm), NAc (bregma 1.845–1.245 mm), MD (bregma −0.59 to −1.79 mm), BLA (bregma −0.59 to −1.79 mm), LH (bregma −0.59 to −1.79 mm), and vCA1 (bregma −2.79 to −3.51 mm; Paxinos and Franklin's The Mouse Brain, 5th edition, 2019). No distinction was made between NAc core and shell. c-Fos^+^ cells were hand-counted using the ImageJ Macro Cell Counter plugin. Positive Fos-like immunoreactivity was indicated by strong cell nuclei staining. Counts were performed by pairs of blinded analyzers, with assignments counterbalanced across brain regions and experimental conditions. Final cell counts were averaged across anterior, medial, and posterior rostrocaudal sections, and an overall mean c-Fos^+^ cell count and density was calculated for each brain region using the area counted in square millimeter.

#### Statistical analysis

Statistical analyses included Shapiro–Wilk tests for normality, Pearson's correlations, and linear regressions to indicate linear fits in scatterplots. Correlations across ranks derived from the urine marking assay, agonistic behaviors during competition, and rewards won during reward competition were assessed using a Pearson's correlation analysis, and *p* values were adjusted with a Bonferroni–Holm multiple-comparison adjustment. Cell counts across brain regions were correlated with the number of port entries in Alone condition (Table 1), mouse weight across all conditions (Table 2), and the number of wins in Competition context (Extended Data Fig. 2-2). Pearson's correlation tests were used, and those *p* values were adjusted using the Benjamini–Hochberg false discovery rate (FDR) procedure. For correlations with percent body weight and cell activity, *p* values were adjusted within experimental conditions (Competition, Together, Alone).

**Table 1.** Pearson's correlations between the mean number of c-Fos^+^ cells and number of port entries during the alone context

Brain region		Data structure	Pearson's *r*	*p* value (two-tailed)	Adjusted *p* value (*q*)	95% confidence interval	*n*
a	ACC	Normal distribution	0.5976	0.0892	0.1465	−0.1103 to 0.9032	9
b	PL	Normal distribution	0.5959	0.0691	0.1465	−0.05401 to 0.8912	10
c	IL	Normal distribution	0.6404	0.0461	0.1465	0.01812 to 0.9051	10
d	NAc	Normal distribution	0.504	0.1665	0.1903	−0.2407 to 0.8752	9
e	MD	Normal distribution	0.4379	0.2057	0.2057	−0.2648 to 0.8368	10
f	BLA	Normal distribution	0.5364	0.1099	0.1465	−0.1408 to 0.8716	10
g	LH	Normal distribution	0.5385	0.1083	0.1465	−0.1379 to 0.8723	10
h	vCA1	Normal distribution	0.669	0.0697	0.1465	−0.06756 to 0.9336	8

*P* values derived from Pearson's correlation analysis were adjusted using the FDR Benjamini–Hochberg procedure. The sample size *n* indicates the number of animals.

**Table 2.** Pearson's correlations between the total number of active cells and percent body weight

Brain region		Context	Data structure	Pearson's *R*	*p* value (two-tailed)	Adjusted *p* value (*q*)	95% confidence interval	*n*
a	ACC	Competition	Normal distribution	0.2017	0.5762	0.8821	−0.4901 to 0.7377	10
b	ACC	Together	Normal distribution	−0.4199	0.3003	0.7193	−0.8678 to 0.4044	8
c	ACC	Alone	Normal distribution	0.7827	0.0126	0.1011	0.2470 to 0.9520	9
d	PL	Competition	Normal distribution	0.1055	0.7718	0.8821	−0.5614 to 0.6893	10
e	PL	Together	Normal distribution	−0.555	0.1533	0.7193	−0.9055 to 0.2458	8
f	PL	Alone	Normal distribution	0.6385	0.0469	0.1878	0.01482 to 0.9045	10
g	IL	Competition	Normal distribution	0.1243	0.7322	0.8821	−0.5482 to 0.6992	10
h	IL	Together	Normal distribution	−0.2637	0.528	0.7193	−0.8166 to 0.5416	8
i	IL	Alone	Normal distribution	0.5346	0.1114	0.2386	−0.1432 to 0.8710	10
j	NAc	Competition	Normal distribution	0.350014	0.3559	0.8821	−0.4080 to 0.8234	9
k	NAc	Together	Normal distribution	−0.0887649	0.8346	0.8346	−0.7457 to 0.6583	8
l	NAc	Alone	Normal distribution	0.0598	0.8785	0.8785	−0.6300 to 0.6957	9
m	MD	Competition	Normal distribution	0.5529	0.1226	0.8821	−0.1758 to 0.8902	9
n	MD	Together	Normal distribution	0.4364	0.3276	0.7193	−0.4717 to 0.8952	7
o	MD	Alone	Normal distribution	0.2772	0.4381	0.5007	−0.4269 to 0.7721	10
p	BLA	Competition	Normal distribution	−0.1586	0.6835	0.8821	−0.7443 to 0.5650	9
q	BLA	Together	Normal distribution	−0.2513	0.5867	0.7193	−0.8445 to 0.6189	7
r	BLA	Alone	Normal distribution	0.4626	0.1782	0.2851	−0.2356 to 0.8459	10
s	LH	Competition	Normal distribution	−0.134	0.7311	0.8821	−0.7329 to 0.5819	9
t	LH	Together	Normal distribution	−0.2471	0.5931	0.7193	−0.8433 to 0.6216	7
u	LH	Alone	Normal distribution	0.5248	0.1193	0.2386	−0.1565 to 0.8677	10
v	vCA1	Competition	Normal distribution	0.0146	0.9753	0.9753	−0.7467 to 0.7593	7
w	vCA1	Together	Normal distribution	−0.2524	0.6294	0.7193	−0.8831 to 0.7032	6
x	vCA1	Alone	Normal distribution	0.4367	0.2794	0.3725	−0.3871 to 0.8728	8

*P* values derived from Pearson's correlation analysis were adjusted using the FDR Benjamini–Hochberg procedure. The sample size *n* indicates the number of animals.

For Figure 2, Type 3 two-way linear mixed-effect ANOVA using Satterthwaite's method was conducted. First a model was conducted on cell densities with brain region (ACC, PL, IL, NAc, MD, BLA, LH, vCA1) and context (Competition, Together, Alone) as fixed effects and mouse ID as random effects. A second model was conducted on the percent change in c-Fos^+^ cell density from the Alone baseline, with context (Competition, Together) and brain region (ACC, PL, IL, NAc, MD, BLA, LH, vCA1) as fixed effects and mouse ID as a random effect. This model tested whether behavioral context modulated deviation from baseline c-Fos activity across brain regions. The percent change from Alone baseline was calculated as follows:

$$\mathrm{percent}\;\mathrm{change}=\frac{\mathrm{regio}n_{i}-{\bar{\mathrm{region}}}_{\mathrm{alone}}}{{\bar{\mathrm{region}}}_{\mathrm{alone}}}\;*\;100.$$

In the above formula, region*_i_* represents the cell density for that region for mouse *i*; ${\bar{\mathrm{region}}}_{\mathrm{alone}}$ represents the estimated marginal mean cell density for that region calculated from the original GLM modeled on cell densities. To visualize “condition effects” on percent changes in c-Fos cell densities, we averaged percent changes in density across all regions per subject for Figure 2*C*. The follow-up two–sample *t* test was conducted on the estimated marginal means of this global c-Fos for each condition after normalization to Alone baseline, namely, Competition and Together. Additionally, one-sample *t* tests (*μ* = 0) were performed to assess whether percent differences from Alone were significantly different from the baseline for each condition across all brain regions. *P* values were corrected for multiple comparisons using the Bonferroni–Holm adjustment for cross condition comparisons (two tests: two conditions) and the Benjamini–Hochberg FDR method for the within each brain region comparison (16 tests, eight brain regions, two conditions). All analyses were conducted in R using the lme4 and emmeans packages.

To analyze functional relationships between brain regions, we used a permutation test (scipy.stats.permutation_test). For each correlation, we generated a null distribution by randomly shuffling cell densities within regions across 2,000 permutations while calculating Pearson's correlation coefficients. For each permutation, subject labels were shuffled within a given brain region, thus maintaining the mean c-Fos values per region. The Benjamini–Hochberg FDR procedure was applied to two-tailed *p* values derived from the permutation test. For cross-regional correlations, FDR adjustments were applied to all brain region pairs within each experimental context separately (Competition, Alone, Together). These values are shown in Tables 3–5. The same permutation procedure was applied to all correlational analyses between dominance measures (relative rank and ELO score) and c-Fos cell counts per brain region. For rank correlations, FDR adjustments were applied within each unique combination of rank metric, assay, and experimental context, correcting across all eight brain regions simultaneously, e.g., all eight regional correlations for relative rank derived from the urine marking assay within the Competition context were adjusted together.

**Table 3.** Pearson's correlation of mean density of c-Fos–active cells across brain regions during Alone context

Brain region A		Brain region B	Data structure	Pearson's *R*	Adjusted *p* value (*q*)	Percentile of observed value in null distribution	*n*
**a**	**ACC**	**BLA**	**Normal distribution**	**0.872**	**0.008**	**99.85**	**9**
**b**	**ACC**	**IL**	**Normal distribution**	**0.875**	**0.0102**	**99.85**	**9**
**c**	**ACC**	**LH**	**Normal distribution**	**0.851**	**0.018**	**99.6**	**9**
d	ACC	MD	Normal distribution	0.589	0.1209	94.65	9
**e**	**ACC**	**PL**	**Normal distribution**	**0.962**	**0.007**	**100**	**9**
f	ACC	NAc	Normal distribution	0.422	0.283	86.4	8
**g**	**ACC**	**vCA1**	**Normal distribution**	**0.783**	**0.0575**	**98.2**	**8**
**h**	**BLA**	**IL**	**Normal distribution**	**0.945**	**0.007**	**100**	**10**
**i**	**BLA**	**LH**	**Normal distribution**	**0.809**	**0.0172**	**99.65**	**10**
**j**	**BLA**	**MD**	**Normal distribution**	**0.752**	**0.0299**	**99.25**	**10**
**k**	**BLA**	**PL**	**Normal distribution**	**0.915**	**0.007**	**100**	**10**
l	BLA	NAc	Normal distribution	0.634	0.0956	95.95	9
**m**	**BLA**	**vCA1**	**Normal distribution**	**0.876**	**0.0117**	**99.8**	**8**
**n**	**IL**	**LH**	**Normal distribution**	**0.871**	**0.0102**	**99.9**	**10**
**o**	**IL**	**MD**	**Normal distribution**	**0.676**	**0.051**	**98.5**	**10**
**p**	**IL**	**PL**	**Normal distribution**	**0.916**	**0.007**	**100**	**10**
q	IL	NAc	Normal distribution	0.686	0.0742	97.4	9
**r**	**IL**	**vCA1**	**Normal distribution**	**0.96**	**0.008**	**99.95**	**8**
**s**	**LH**	**MD**	**Normal distribution**	**0.738**	**0.0315**	**99.15**	**10**
**t**	**LH**	**PL**	**Normal distribution**	**0.874**	**0.008**	**99.95**	**10**
u	LH	NAc	Normal distribution	0.47	0.197	90.9	9
**v**	**LH**	**vCA1**	**Normal distribution**	**0.898**	**0.0102**	**99.85**	**8**
w	MD	PL	Normal distribution	0.643	0.076	97.2	10
x	MD	NAc	Normal distribution	0.284	0.4308	78.5	9
**y**	**MD**	**vCA1**	**Normal distribution**	**0.712**	**0.0663**	**97.8**	**8**
z	PL	NAc	Normal distribution	0.62	0.0956	95.95	9
**aa**	**PL**	**vCA1**	**Normal distribution**	**0.866**	**0.0102**	**99.85**	**8**
ab	NAc	vCA1	Normal distribution	0.752	0.0956	96.15	7

Adjusted *p* values were derived from a permutation distribution of Pearson's correlation coefficients and then adjusted using the FDR Benjamini–Hochberg procedure. Rows in gray indicate significant correlations after correction. Percentile values show where each observed correlation falls within the null distribution of the permutation test. Bolded rows indicate correlations in the top/bottom 2.5% of null distribution. *n*, number of paired samples (subjects) per calculation.

**Table 4.** Pearson's correlation of mean density of c-Fos–active cells across brain regions during Together context

Brain region A		Brain region B	Data structure	Pearson's *R*	Adjusted *p* value (*q*)	Percentile of observed value in null distribution	*n*
a	ACC	BLA	Normal distribution	−0.309	0.637	23.85	7
b	ACC	IL	Normal distribution	0.208	0.7395	67.2	8
c	ACC	LH	Normal distribution	0.518	0.4878	87.85	7
d	ACC	MD	Normal distribution	0.095	0.907	56.3	7
e	ACC	PL	Normal distribution	0.793	0.4303	96	8
f	ACC	NAc	Normal distribution	0.632	0.4303	96.15	8
g	ACC	vCA1	Normal distribution	−0.234	0.7395	32.05	6
h	BLA	IL	Normal distribution	0.753	0.4303	96.95	7
i	BLA	LH	Normal distribution	0.532	0.4844	89.7	7
j	BLA	MD	Normal distribution	0.177	0.7395	65.75	7
k	BLA	PL	Normal distribution	0.643	0.4303	94.3	7
l	BLA	NAc	Normal distribution	0.284	0.6653	73.9	7
**m**	**BLA**	**vCA1**	**Normal distribution**	**0.9**	**0.4303**	**98.7**	**6**
n	IL	LH	Normal distribution	0.652	0.4303	93.9	7
o	IL	MD	Normal distribution	−0.04	0.9745	48.7	7
p	IL	PL	Normal distribution	0.411	0.4915	86.05	8
q	IL	NAc	Normal distribution	0.275	0.637	76.5	8
r	IL	vCA1	Normal distribution	0.456	0.5367	83.93	6
s	LH	MD	Normal distribution	0.516	0.4844	88.85	7
t	LH	PL	Normal distribution	0.63	0.4303	94.58	7
u	LH	NAc	Normal distribution	0.594	0.4447	92.95	7
v	LH	vCA1	Normal distribution	0.5	0.5426	82.8	6
w	MD	PL	Normal distribution	0.183	0.7395	66.7	7
x	MD	NAc	Normal distribution	0.555	0.4844	88.8	7
y	MD	vCA1	Normal distribution	0.444	0.5936	79.9	6
z	PL	NAc	Normal distribution	0.513	0.4844	90.45	8
aa	PL	vCA1	Normal distribution	0.725	0.4303	94.4	6
ab	NAc	vCA1	Normal distribution	0.513	0.4915	87.25	6

Adjusted *p* values were derived from a permutation distribution of Pearson's correlation coefficients and then adjusted using the FDR Benjamini–Hochberg procedure. Percentile values show where each observed correlation falls within the null distribution of the permutation test. Bolded rows indicate correlations in the top/bottom 2.5% of null distribution. *n*, number of paired samples per calculation.

**Table 5.** Pearson's correlation of mean density of c-Fos–active cells across brain regions during Competition context

Brain region A		Brain region B	Data structure	Pearson's *R*	Adjusted *p* value (*q*)	Percentile of observed value in null distribution	*n*
a	ACC	BLA	Normal distribution	−0.549	0.4958	5.85	9
b	ACC	IL	Normal distribution	0.43	0.6682	90.5	10
c	ACC	LH	Normal distribution	−0.64	0.2869	2	9
d	ACC	MD	Normal distribution	0.148	0.9569	65.7	9
e	ACC	PL	Normal distribution	0.545	0.4958	95.1	10
f	ACC	NAc	Normal distribution	0.268	0.9569	75.15	9
g	ACC	vCA1	Normal distribution	−0.217	0.9569	33	7
h	BLA	IL	Normal distribution	−0.432	0.8396	13.45	9
**i**	**BLA**	**LH**	**Normal distribution**	**0.876**	**0.1679**	**99.75**	**9**
j	BLA	MD	Normal distribution	0.149	0.9569	65.1	9
k	BLA	PL	Normal distribution	−0.219	0.9569	28.9	9
l	BLA	NAc	Normal distribution	−0.317	0.9569	18.45	9
m	BLA	vCA1	Normal distribution	−0.099	0.9783	40.75	7
n	IL	LH	Normal distribution	−0.277	0.9569	22.05	9
o	IL	MD	Normal distribution	−0.218	0.9569	26.85	9
**p**	**IL**	**PL**	**Normal distribution**	**0.765**	**0.2099**	**99.3**	**10**
**q**	**IL**	**NAc**	**Normal distribution**	**0.741**	**0.2146**	**98.9**	**9**
r	IL	vCA1	Normal distribution	0.004	0.9815	49.05	7
s	LH	MD	Normal distribution	−0.021	0.9815	48.35	9
t	LH	PL	Normal distribution	−0.142	0.9569	34.2	9
u	LH	NAc	Normal distribution	−0.069	0.9783	41.9	9
v	LH	vCA1	Normal distribution	0.275	0.9569	74	7
w	MD	PL	Normal distribution	0.1	0.9783	58.8	9
x	MD	NAc	Normal distribution	−0.146	0.9569	35.85	9
y	MD	vCA1	Normal distribution	−0.094	0.9815	47.9	7
z	PL	NAc	Normal distribution	0.559	0.4958	93.85	9
aa	PL	vCA1	Normal distribution	−0.28	0.9569	28.4	7
ab	NAc	vCA1	Normal distribution	0.05	0.9815	54.35	7

Adjusted *p* values were derived from a permutation distribution of Pearson's correlation coefficients and then adjusted using the FDR Benjamini–Hochberg procedure. Percentile values show where each observed correlation falls within the null distribution of the permutation test. Bolded rows indicate correlations in the top/bottom 2.5% of null distribution. *n*, number of paired samples per calculation.

Behavior schematics were created with BioRender.com.

## Results

### Competitive outcomes are independent of baseline reward learning performance

To examine how social context shapes neural responses to reward, we compared male mice across three conditions: Competition (mice competed for cued rewards against a cagemate), Together (mice received cued rewards in a noncompetitive social setting), and Alone (mice received rewards without a social partner; Fig. 1*A*,*B*). Social ranks were independently assessed based on reward competition outcomes, agonistic behaviors displayed during reward competition, and territory-based markings, resulting in reward-based ranks, agonistic-based ranks, and territory-based ranks. Notably, the urine marking rank was not significantly correlated with the reward competition rank (*r*_(25)_ = 0.1700; *p* = 0.7930; Fig. 1*Ci*) or the agonistic-based rank (*r*_(25)_ = 0.1093; *p* = 0.7930; Fig. 1*Ciii*), suggesting that these measures capture distinct aspects of social hierarchy. Trending correlations were seen between agonistic-based and reward-based dominance ranks (*r*_(25)_ = 0.4497; *p* = 0.0558; Fig. 1*Cii*). This trend aligns with the concurrent measurement of both behaviors during reward competitions, where agonistic displays and competitive success likely interact dynamically. Importantly, reward competition rank did not correlate with latency to retrieve the reward on the ninth training day, suggesting that individual differences in competitive success were not driven by baseline reward learning ability (Fig. 1*D*). To assess how reward context influences neural activity in prefrontal and subcortical regions, mice from the Competition, Together, and Alone groups were perfused after the final reward exposure.

### Reward context impacts neural activity across prefrontal and subcortical regions

To assess how reward context influences neural activity in prefrontal and subcortical circuits, we compared mean c-Fos^+^ cell densities across the Competition, Together, and Alone conditions (Fig. 2*A*,*B*). We found a significant difference in c-Fos expression across brain regions (*F*_(7, 158.289)_ = 19.5113; *p* < 0.0001), but no effect of reward context (*F*_(2, 24.607)_ = 2.6438; *p* = 0.0911) nor a significant interaction between region and context (*F*_(14, 158.289)_ = 1.1756; *p* = 0.2988) was observed. Furthermore, we wanted to assess whether potential c-Fos differences in the Competition context were driven by the number of rewards given as this would potentially obfuscate further activation pattern assessments within the Competition context. We found no correlation between c-Fos activity and the number of rewards won across all regions (Extended Data Fig. 2-2). Given that our primary goal was investigating the effects of reward context in social settings, we opted to use Alone c-Fos levels of activation as a baseline to facilitate interpretation of social context-driven changes. We normalized c-Fos activity in social contexts (Together and Competition) for each brain region by calculating the change in density of c-Fos^+^ cells compared with the average cell density in the Alone context. A mixed-model ANOVA revealed a significant main effect of condition on normalized c-Fos activity across regions (*F*_(1, 16.057)_ = 7.6011; *p* = 0.0140), with no significant effect of region nor the interaction between the two. Furthermore, the Together context showed significantly greater global c-Fos activation (averaged across regions) than both the Alone context baseline (*t*_(15.7)_ = 3.398; *p* = 0.0075) and the Competition context (*t*_(15.9)_ = −3.207; *p* = 0.0055; Fig. 2*C*,*D*). However, Competition c-Fos levels did not significantly change from Alone baseline levels (*t*_(16.0)_ = −1.019; *p* = 0.3232; Fig. 2*C*; Extended Data Fig. 2-3). Together, these findings suggest that social context globally influences c-Fos activation in these prefrontal and subcortical regions.

**Figure 3.**
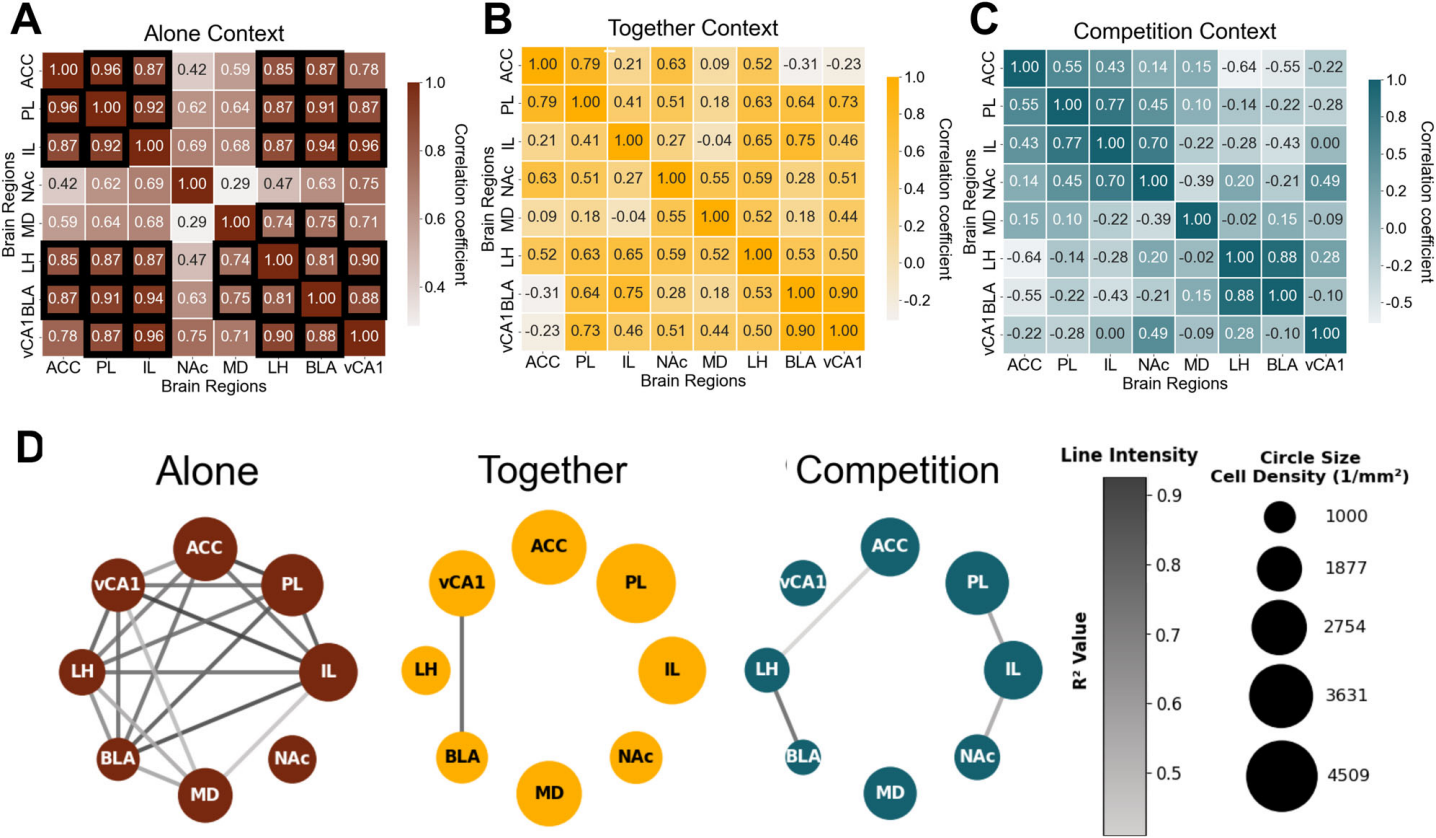
Network-level correlations depend on the reward context. Correlation of cell densities across brain regions during the (***A***) Alone context, (***B***) Together context, or (***C***) Competition context. Each square in the heatmaps displays the Pearson's correlation coefficient (*r*). *P* values derived from permutation testing and then adjusted using Benjamini–Hochberg FDR procedure. Region pairs with black outlines are significantly correlated (*p* < 0.05 after FDR). Statistical details and sample size in Tables 3–5. ***D***, Network visualization showing strong correlations for each context. The circle size represents mean cell density per region. Line intensity represents correlation strength (*R*^2^) between connected regions. Only connections with correlations in the top/bottom 2.5% of null distribution are shown in ***D***. Legends show line intensity for correlation strength (*R*^2^ values) and example circle sizes with corresponding cell density values (cells per square millimeter).

10.1523/ENEURO.0158-25.2025.f2-3 Figure 2-3 **Individual brain regions c-Fos densities normalized to Alone baseline.** (**A-H**) Percent differences in c-Fos⁺ cell density calculated from Alone baseline across conditions for each brain region. Each data point represents the difference from baseline for an individual subject for that brain region. Positive values indicate increased activity relative to Alone; negative values indicate decreased activity. Box plots show median and interquartile ranges. Download Figure 2-3, TIF file.

### Social context—not reward-seeking behavior or hunger—drives neural activity patterns

We examined other potential nonsocial factors that could explain c-Fos activation levels. First, we used port entries as a proxy for reward motivation levels when the animals were alone. No region had significant correlations between c-Fos^+^ counts and the number of port entries when rewards were given to the animal alone (Table 1), suggesting that reward-seeking behavior alone did not drive overall c-Fos activation. Given that hunger may influence the perceived value of rewards, we quantified body weight change as a percentage of prefood restriction weights. After accounting for multiple comparisons, we saw no significant correlations between percent body weight and c-Fos activation (Table 2). These results suggest that regional differences observed across reward social contexts are not solely due to hunger levels or differences in reward behavior but rather induced by differences in the social context.

### Cross-regional correlations change depending on the social reward context

Given that we observed a global effect of social context on c-Fos activation, we next considered how functionally connected (i.e., coactivated) these regions were during the three reward contexts. As a proxy for functional connectivity, we assessed cross-correlations between c-Fos^+^ density across brain regions in the Competition, Together, and Alone contexts. Several studies have validated this cross-correlation approach by quantifying c-Fos across multiple brain regions and their subdivisions (Wheeler et al., 2013; Vetere et al., 2017). To assess the significance of our cross-correlation results, we permutated the data 2,000 times, calculated *p* values based on this null distribution, and performed multiple-comparison adjustments. This analysis revealed that each social context had distinct patterns of correlations (Fig. 3*A*–*C*; Table 3). We found that when mice received rewards in the Alone context, there was widespread cross-regional correlation (Fig. 3*A*), suggesting coordinated activation across the network during reward processing in isolation. Unlike the extensive cross-regional correlations in the Alone context, the social contexts exhibited much fewer cross-regional correlations (Fig. 3*B*,*C*). Next, we explored which correlations differed from chance by identifying those correlation values that were above 97.5th or below 2.5th percentiles. During the Together condition, BLA-vCA1 showed a positive correlation above the 97.5th percentile of a null distribution (Fig. 3*D*; Table 4). In contrast, during Competition context, BLA-LH, IL-PL, and IL-NAc showed positive correlation coefficients above the 97.5th percentile, and ACC-LH showed a strong negative correlation coefficient below the 2.5th percentile of a null distribution (Fig. 3*D*; Table 5). These results suggest potential regional associations for future investigation. Taken together, these findings show that social reward contexts, whether competitive or not, reshape functional coordination across prefrontal and subcortical regions, in comparison with nonsocial reward contexts, as reflected by distinct c-Fos correlation patterns.

### Correlations between dominance rank and prefrontal activity depend on the context

To investigate how social hierarchy shapes reward processing across competitive and noncompetitive contexts, we analyzed the relationship between social ranks and c-Fos^+^ cells. We calculated social ranks based on three ethologically distinct dominance measures: a reward-based social rank using outcomes of the reward competition, a territorial marking-based social rank using urine marking behavior, and an agonistic-based social rank using agonistic behaviors during the reward competition matches.

**Figure 4.**
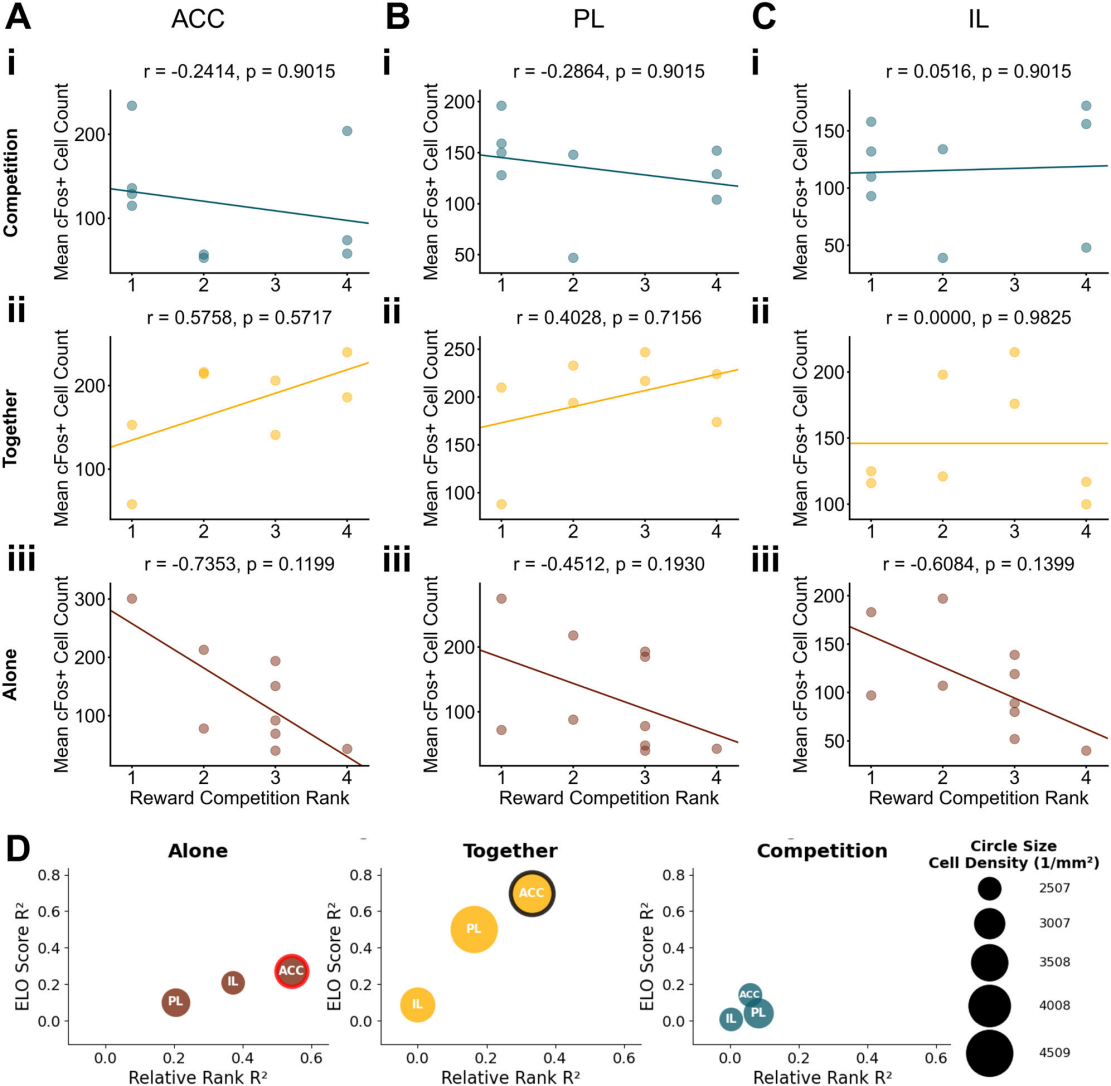
Correlations between reward-based social rank and mPFC activity change depending on the reward context. Correlations between reward competition rank score and neural activity in the (***A***) ACC, (***B***) PL, and (***C***) IL during (***i***) Competition context, (***ii***) Together context, and (***iii***) Alone context. Rank 1 indicates the highest relative dominance. Each dot represents an individual animal, with lines of best fit derived from a linear regression analysis. *P* values were adjusted using a Benjamini–Hochberg FDR procedure. ***D***, A bubble plot summarizing correlations between reward competition dominance metrics and c-Fos^+^ cell counts across brain regions. Each bubble represents one brain region in one context. The circle size represents mean cell density per region. *X*-axis shows correlation strength (*R*^2^) between relative rank and cell count; *Y*-axis shows *R*^2^ values between Elo score and cell count. Colored outlines indicate correlations in the top/bottom 2.5% of null distribution: red outlines for relative rank correlations and black outlines for Elo score correlations. Legend shows example circle sizes with corresponding cell density values (cells/per square millimeter).

**Figure 5.**
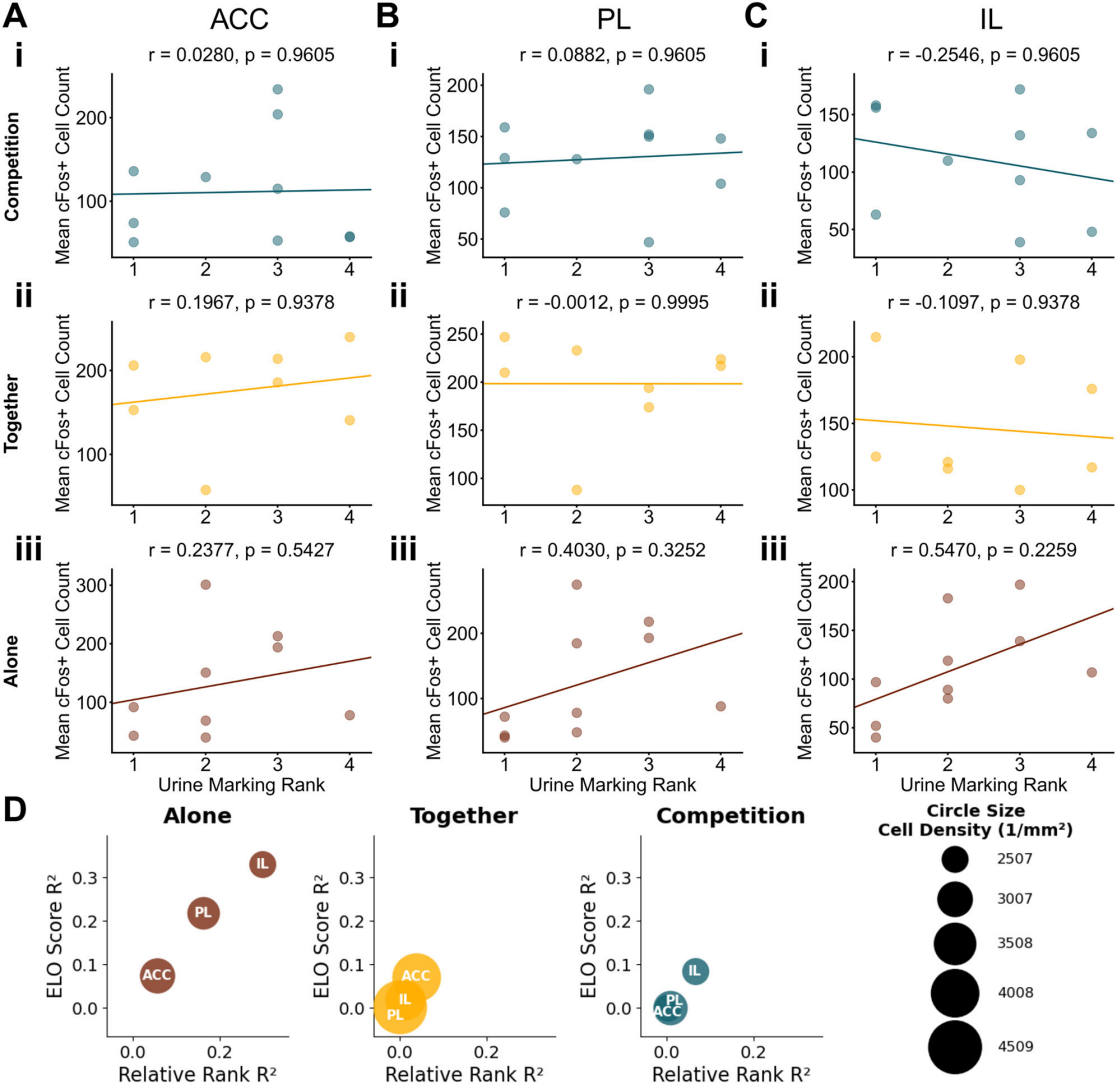
Correlations between territorial marking-based social rank and mPFC activity. Correlations between urine marking rank score and neural activity in the (***A***) ACC, (***B***) PL, and (***C***) IL during (***i***) Competition context, (***ii***) Together context, and (***iii***) Alone context. Rank 1 indicates highest relative dominance. Each dot represents an individual animal, with lines of best fit derived from a linear regression analysis. *P* values were adjusted using a Benjamini–Hochberg FDR procedure. Pearson's *r* and the corresponding FDR *p* value shown in each subplot. Individual dots are partially transparent to show overlapping datapoints. ***D***, A bubble plot summarizing correlations between territorial dominance metrics and c-Fos^+^ cell counts across brain regions. Each bubble represents one brain region in one context. The circle size represents mean cell density per region. *X*-axis shows correlation strength (*R*^2^) between relative rank and cell count; *Y*-axis shows *R*^2^ values between the Elo score and cell count. Colored outlines indicate correlations in the top/bottom 2.5% of null distribution: red outlines for relative rank correlations and black outlines for Elo score correlations. Legend shows example circle sizes with corresponding cell density values (cells/per square millimeter).

**Figure 6.**
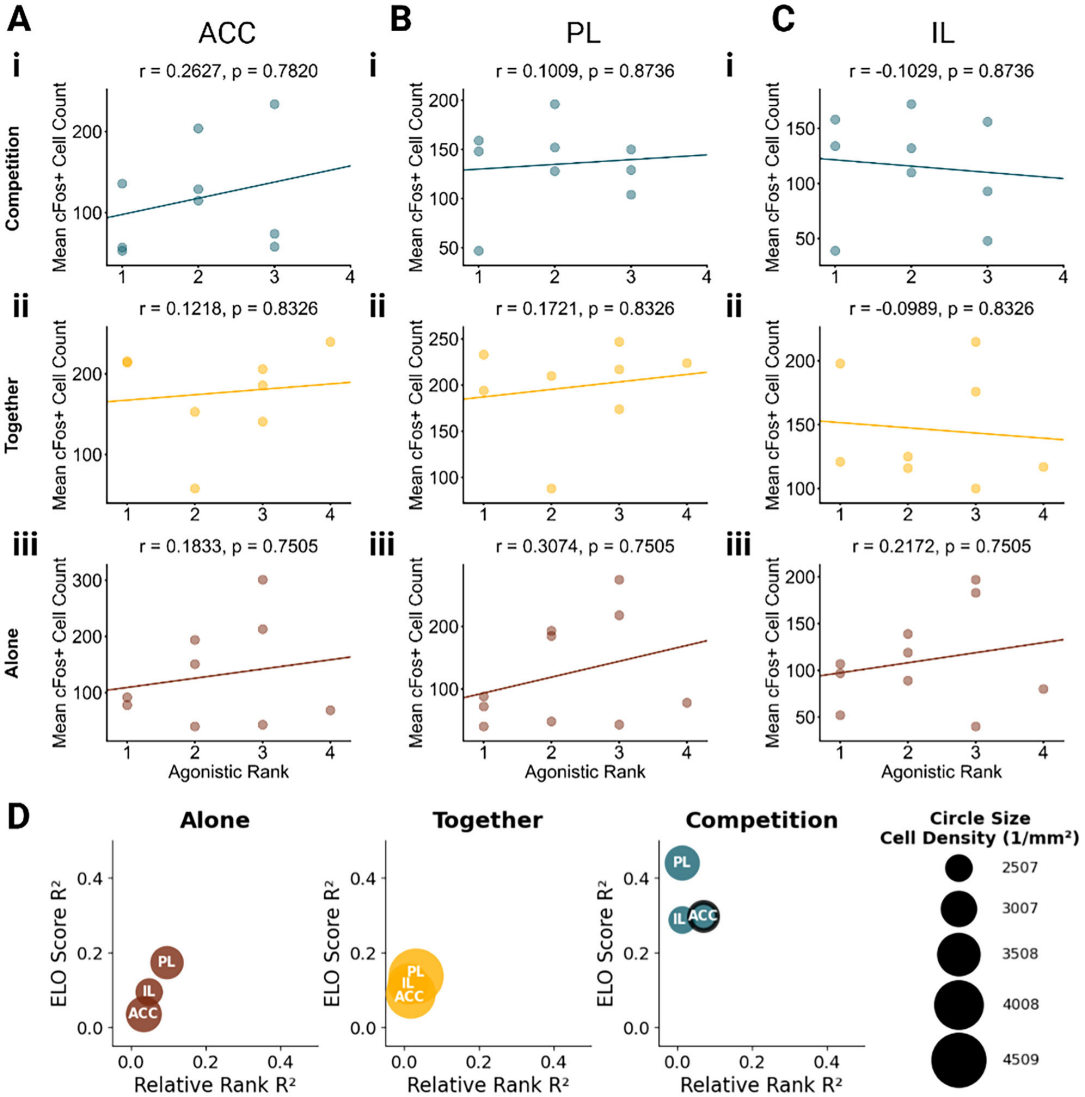
Correlations between agonistic-based social rank and mPFC activity. Correlations between agonistic-based rank score and neural activity in the (***A***) ACC, (***B***) PL, and (***C***) IL during (***i***) Competition context, (***ii***) Together context, and (***iii***) Alone context. Rank 1 indicates highest relative dominance. Each dot represents one animal, with lines of best fit derived from a linear regression analysis. *P* values were adjusted using a Benjamini–Hochberg FDR procedure. ***D***, A bubble plot summarizing correlations between agonistic dominance metrics and c-Fos^+^ cell counts across brain regions. *X*-axis shows correlation strength (*R*^2^) between relative rank and cell count; *Y*-axis shows *R*^2^ values between Elo score and cell count. Black outline indicates correlation in the top/bottom 2.5% of null distribution for the Elo score. Legend shows example circle sizes with corresponding cell density values (cells/per square millimeter).

We next examined correlations between c-Fos^+^ cell counts in mPFC subregions and social ranks. Importantly, most correlations between rank and c-Fos expression were similar whether we used relative rank or dominance scores (Elo scores; Figs. 4–6*D*). Although there were no significant correlations between all social ranks and mPFC c-Fos after adjusting for multiple comparisons (Figs. 4–6), the correlation between reward-based dominance and ACC activity during the Alone context was below the 2.5th percentile of a null distribution (*r* = −0.7353; *p* = 0.1199; Fig. 4*Aiii*), suggesting that higher-ranking mice may show higher activity than subordinates (Fig. 4*D*). Overall, these results suggest that these three social rank measures capture distinct dimensions of dominance with different relevance to neural activity in the mPFC when processing rewards. Thus, social dominance, as measured by reward competition but not territorial marking or aggression, impacts how the prefrontal cortex processes rewards obtained in isolated contexts.

### Correlations between dominance rank and subcortical activity depend on the context

**Figure 7.**
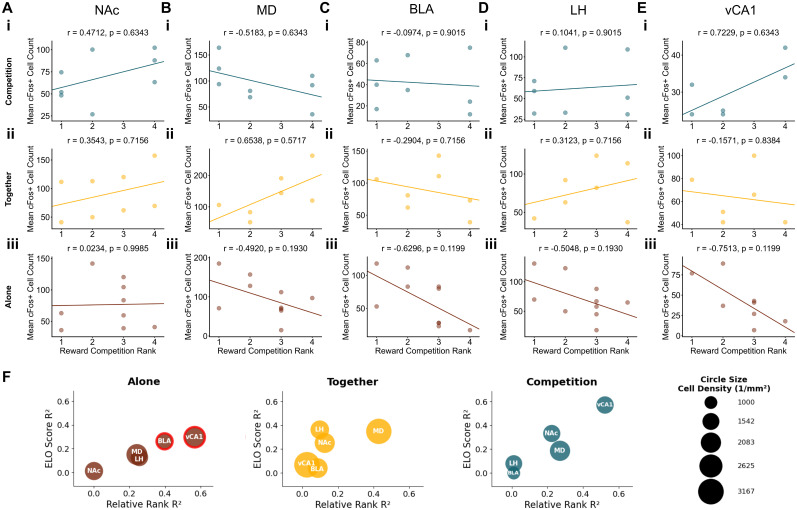
Correlations between reward-based social rank and subcortical activity across reward contexts. Correlations between reward competition rank and neural activity in the (***A***) NAc, (***B***) MD, (***C***) BLA, (***D***) LH, and (***E***) vCA1 during (***i***) Competition context, (***ii***) Together context, and (***iii***) Alone context. Rank 1 indicates highest relative dominance. Each dot in the scatterplot represents an individual animal, with lines of best fit derived from a linear regression analysis. Pearson's *r* and corresponding *p* value shown in each subplot. *P* values were adjusted using a Benjamini–Hochberg FDR procedure. ***F***, A bubble plot summary showing correlations between reward competition dominance metrics and c-Fos^+^ cell counts across brain regions. *X*-axis shows correlation strength (*R*^2^) between relative rank and cell count; *Y*-axis shows *R*^2^ values between Elo score and cell count. Red outlines indicate correlations in the top/bottom 2.5% of null distribution for relative rank correlations. Legend shows example circle sizes with corresponding cell density values (cells/per square millimeter).

**Figure 8.**
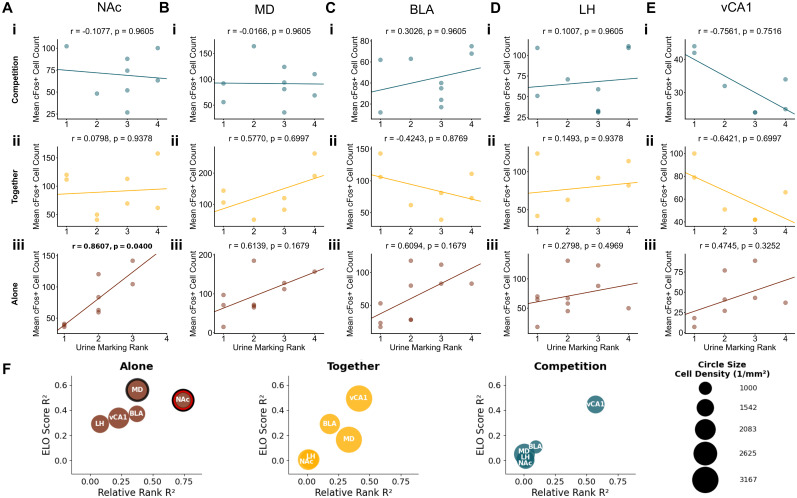
Correlations between territorial marking-based social rank and subcortical activity across reward contexts. Correlations between urine marking rank score and neural activity in (***A***) NAc, (***B***) MD, (***C***) BLA, (***D***) LH, and (***E***) vCA1 during (***i***) Competition context, (***ii***) Together context, and (***iii***) Alone context. Rank 1 indicates highest relative dominance. Each dot in the scatterplots represents an individual animal, with lines of best fit derived from a linear regression analysis. *P* values were adjusted using a Benjamini–Hochberg FDR procedure. Significant FDR *p* values are in bold. ***F***, A bubble plot summarizing correlations between territorial dominance metrics and c-Fos^+^ cell counts across brain regions. *X*-axis shows correlation strength (*R*^2^) between relative rank and cell count; *Y*-axis shows *R*^2^ values between the Elo score and cell count. Colored outlines indicate correlations in the top/bottom 2.5% of null distribution: red outlines for relative rank correlations and black outlines for Elo score correlations. Legend shows example circle sizes with corresponding cell density values (cells/per square millimeter).

**Figure 9.**
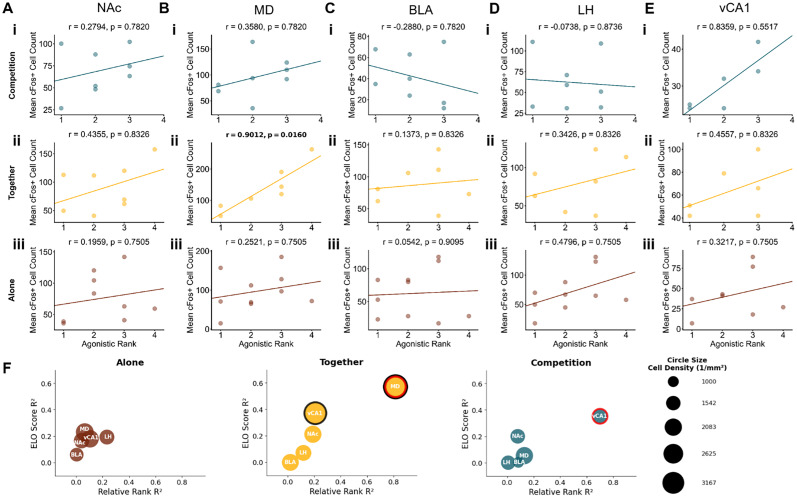
Correlations between agonistic-based social rank and subcortical activity across reward contexts. Correlations between agonistic rank score and neural activity in (***A***) NAc, (***B***) MD, (***C***) BLA, (***D***) LH, and (***E***) vCA1 during (***i***) Competition context, (***ii***) Together context, and (***iii***) Alone context. Rank 1 indicates highest relative dominance. Each dot in the scatterplots represents an individual animal, with lines of best fit derived from a linear regression analysis. *P* values were adjusted using a Benjamini–Hochberg FDR procedure. ***F***, A bubble plot summarizing correlations between agonistic dominance metrics and c-Fos^+^ cell counts across brain regions. *X*-axis shows correlation strength (*R*^2^) between relative rank and cell count; *Y*-axis shows *R*^2^ values between the Elo score and cell count. Colored outlines indicate correlations in the top/bottom 2.5% of null distribution: red outlines for relative rank correlations, black outlines for Elo score correlations. Legend shows example circle sizes with corresponding cell density values (cells/per square millimeter).

To explore how dominance rank impacts subcortical neural activity across reward contexts, we analyzed the correlations between our three dominance measures and c-Fos^+^ cells in the subcortical regions during Competitive, Together, and Alone contexts. Notably, our most robust effects for territorial and reward-based ranks emerged during the Alone context rather than during competition, even though social hierarchies may seem most relevant in competitive settings (Figs. 7–9*F*). Correlations between reward-based rank and neural activity in both the BLA and vCA1 fell below the 2.5th percentile threshold (BLA, *r* = −0.6296; *p* = 0.1199; vCA1, *r* = −0.7513; *p* = 0.1199; Fig. 7), suggesting dominant mice exhibited higher neural activation in these regions during isolated reward experiences. In the Alone context, NAc activity was negatively correlated with territorial-based dominance (*r* = −0.8607; *p* = 0.0400; Fig. 8*Aiii*), indicating that subordinate mice showed higher NAc activation when experiencing rewards alone. On the other hand, agonistic-based rankings were more relevant during social contexts. In the Together context, subordinate mice showed greater MD activity than dominant mice based on agonistic-based rankings (*r* = −0.9012; *p* = 0.0160; Fig. 9*Bii*). In the Competition context, although not significant, the correlation between vCA1 activity and agonistic rank exceeded the 97.5th percentile (*r* = 0.8359; *p* = 0.5517; Fig. 9*Ei*), with subordinate mice showing higher vCA1 activity than dominant mice. Overall, these results further support that distinct domains of social dominance modulate distinct pathways in the reward processing neural systems.

### Performance on dominance-based rank assessments is unaffected by hunger levels

To validate our social rank measures for the competition assay, we investigated whether competitive performance could be explained by other nonsocial variables. There were no significant correlations between percent body weight and reward competition social rank score (*r*_(25)_ = −0.0135; *p* = 0.9468), indicating that hunger levels did not drive outcomes of the competition. Similarly, percent body weight did not correlate with territorial marking-based social rank scores (*r*_(26)_ = −0.0090; *p* = 0.9639). Overall, our results show that social hierarchy affects reward-related neural activity, but these effects depend on both the reward's context and which aspect of social dominance is being measured.

## Discussion

Our study reveals that the context of reward greatly impacts network activation. Notably, the consumption of a reward when alone initiated network-wide correlations across regions, while rewards experienced socially, both in competitive and noncompetitive states, markedly reduced these correlations. Furthermore, while social rank marginally impacted c-Fos activation during competitive reward contexts, it significantly impacts neural activity when animals process rewards in isolated and socially noncompetitive states. These findings provide critical insights into the neural mechanisms mediating reward-based social competition. Below, we discuss our observations while considering the exploratory nature of our study and the limitations in the sample size.

### Social context impacts the whole network and reshapes functional connectivity patterns

Our results reveal a striking reorganization of neural correlation patterns depending on social context. In the Alone context, we observed widespread correlations across multiple brain regions, suggesting high functional synchrony across these regions during nonsocial, noncompetitive reward processing. This widespread connectivity is markedly reorganized when social or competitive elements are introduced, as evidenced by the almost complete lack of regional correlations in the Competition and Together contexts. Despite similar network decorrelation in both Competition and Together contexts, social context significantly affected global c-Fos activation levels. Experiencing rewards in a social noncompetitive context evoked the largest activation throughout all regions measured. Conversely, the Competition context did not show an increase in c-Fos activation, even though it showed a marked reduction of cross-regional correlations compared with the Alone context. These results suggest that noncompetitive social reward interactions recruit larger neural ensembles in this region or that competition induces local inhibition. This enhanced activation may be driven by the combined rewarding effects of the social and liquid reward stimuli, whereas during competition, the social stimulus may become less rewarding when it represents a competitor. Alternatively, social competition may trigger increased local inhibitory activity within the mPFC circuits. This latter interpretation aligns with recent evidence demonstrating that mPFC interneuron activity plays a crucial role in driving winning behavior during social competition (Zhang et al., 2022). The PL and ACC, but not IL, exhibited a tendency to be recruited when mice obtained rewards in social contexts. This result is consistent with previous reports, which have demonstrated that these regions regulate behaviors during reward competition (Li et al., 2022; Padilla-Coreano et al., 2022). The network decorrelation exhibited during Competition could further reflect the adaptive resource allocation needed to meet the demands of increasingly complex social environments.

### Relationship between dominance ranks and neural activity across reward contexts

To evaluate the significance of the correlations between c-Fos activity and social rank, we used a nonparametric, percentile-based method that compares observed correlation coefficients to those in a null distribution generated through permutation. This approach has been successfully applied in previous brain-wide c-Fos studies in rodents (Kim et al., 2016). Using this method, we found correlations between c-Fos activation and social rank in specific contexts, with preliminary evidence suggesting that cortical and subcortical activity patterns differ by the type of social rank test used and reward context. Preliminary patterns suggested that dominant mice, based on the reward competition rank, may show higher activity in ACC, BLA, and vCA1 when rewards were pursued in nonsocial contexts. Given that both ACC and BLA regions regulate dominance behaviors (Li et al., 2022; Scheggia et al., 2022), this pattern suggests that dominant individuals may engage both regions to process reward valuation or maintain goal-directed behavior even in the absence of social competition. Although not statistically significant after multiple-comparison correction, ACC activity showed the strongest positive correlation with competitive success as measured by the number of rewards won (*r* = 0.88), suggesting this relationship warrants further investigation using larger samples. Previous work demonstrates that ACC silencing decreases reward acquisition during social competition (Li et al., 2022). A correlation between ACC activity and competitive success aligns with the region's established role in effort-based decision-making and highlights its specific contribution to competitive reward acquisition. Interestingly, a study in nonhuman primates demonstrated that BLA neurons that encode social rank overlap with those that encode reward (Munuera et al., 2018). In contrast, despite a recent study showing that NAc activity is necessary to express dominance in the tube test (Shan et al., 2022), our results showed that NAc c-Fos activity was negatively correlated with dominance as measured through territory marking in the Alone context. This result suggests that in a noncompetitive context, dominance-related activity could be inhibited when mice pursue a reward in an isolated environment.

Further context-dependent neural activity was observed in the hippocampus. We found that vCA1 activity correlated with reward-based rank in the Alone context and with agonistic-based rank in the Competition context. These preliminary findings suggest that vCA1 may integrate social and motivational cues in a context-specific manner. The observed pattern of vCA1 activity in the Alone context, in contrast to its pattern with agonistic rank in a competitive context, highlights vCA1 potential sensitivity to the context. In the Alone context, the reward remains the most salient stimulus, while in the Competition context, the competitor must also be accounted for. This aligns with recent findings that vCA1 neurons encode the conjunction of social stimuli, their spatial location, and environmental context (Wu et al., 2023), supporting the idea that vCA1 plays a key role in integrating social experience with contextual cues. Moreover, the relationships between neural activity and rank were highly specific to a particular dominance metric, with territorial-based rank affecting NAc activity, agonistic-based rank modulating MD activity, and reward-based ranks influencing BLA and ACC responses—demonstrating that distinct facets of dominance engage different neural circuits in context-specific ways. These findings suggest that the impacts of social rank on neural activity persists beyond competitive contexts, with dominance influencing reward processing through distinct mechanisms that vary by the hierarchy type and social context. Taken together, these results suggest that not only does the context modulate how dominance impacts reward responses, but that dominance hierarchies most strongly influence subcortical reward processing when mice experienced rewards alone. This pattern raises some consideration for whether reward processing capabilities shape social rank or established social rank modulates reward processing. By incorporating complementary dominance assays, our study demonstrates that different dimensions of social hierarchy uniquely shape neural activity in the mPFC, with competition-derived dominance exerting stronger influence on reward response modulation than territorial dominance.

### Considerations and future directions

An important follow-up question to this study is whether the same circuits are also involved in reward-based social competition in female mice. The current literature on neural mechanisms for reward competition has been done exclusively in male mice (Li et al., 2022; Padilla-Coreano et al., 2022; Cum et al., 2024), and territorial urine marking has only been seen in breeding or wild female mice (Maruniak et al., 1975; Hurst, 1990). Much work needs to be done to assess what dominance-based assays most reliably measure dominance in females, including the reward competition assay. Additionally, it has been shown that there are sex differences in how social and nonsocial rewards are encoded in the prefrontal cortex (Isaac et al., 2024). Differential reward encodings plus ambiguous dominance metrics within female mice make the sexual dimorphisms of reward and competition encoding and important gap to be address by future studies.

It is also important to note that the absence of differential c-Fos expression in some regions does not preclude their involvement in social competition. The limitations of c-Fos as an activity marker, specifically capturing only a single time point without cell-type specificity, may obscure more nuanced activity patterns. For instance, D1 and D2 subpopulations in NAc play opposing roles in social dominance (Shan et al., 2022). Thus, distinct populations of neurons within the NAc are not discernible using general c-Fos labeling. Future studies employing temporally precise techniques, such as electrophysiology or calcium imaging, will provide more detailed characterization of these regions’ contributions to reward-based competition. In addition, circuit-level manipulations, such as optogenetics manipulations, of specific subpopulations will provide important knowledge regarding the causal role of circuit activity in driving reward-seeking behavior in social contexts.

Given the exploratory nature of this study examining the effects of multiple social rank metrics across brain regions and the limits of our sample size, we used permutation testing to identify rare correlations compared with a null distribution. While many of these correlations, particularly in the Together and Competition conditions, were no longer significant after multiple-comparison correction, we chose to report them in the main text to provide transparency in this exploratory analysis and minimize the risk of false negatives in an underpowered study. This work establishes initial evidence that can guide the selection of brain regions and contexts for future validation studies. Future studies are needed to clarify the precise nature of these functional relationships and delineate how specific neuronal populations within these regions orchestrate the complex behaviors underlying social competition for rewards.

## Conclusion

Our findings highlight circuits that may be critical for mediating reward-based social competition. The context and social rank-dependent engagement and coordinated activity within these circuits provide a foundation for understanding the neural basis of competitive social interactions. Finally, because disruptions in reward processing and social cognition are implicated in multiple neuropsychiatric disorders, these findings provide important considerations for translational studies in models for neuropsychiatric disorders.

## Synthesis

Reviewing Editor: Sam Golden, The University of Washington Decisions are customarily a result of the Reviewing Editor and the peer reviewers coming together and discussing their recommendations until a consensus is reached. When revisions are invited, a fact-based synthesis statement explaining their decision and outlining what is needed to prepare a revision will be listed below. The following reviewer(s) agreed to reveal their identity: NONE.

Dear Dr. ,

Your manuscript has been reviewed by two experts in the field. While both find the manuscript of interest and import, there are shared major concerns with the statistical approach used. Through discussion, the reviewers no longer have concerns about the correlational nature of the observations, so while you may and should address these concerns in your rebuttal, it will not factor into the final editorial decision (as eNeuro welcomes such manuscripts).

Reviewer 1:

In this article, authors use c-FOS labeling to systematically map how reward responses change across social contexts. They find intriguing differences in which subregions are functionally correlated depending on social context and social rank. Intriguingly, they find significant differences in how activity in several regions in the social decision making network correlate with reward-based rank vs territory-based ranking of social hierarchy. Additionally, they find no correlation between social ranks generated by these two methods. This is a particularly interesting finding that informs our understanding of social group dynamics and the validity of these different social dominance assays. The authors should consider expanding on this theme in the discussion. While the paper has several interesting findings, there are some significant concerns regarding the methodological details and the interpretations of findings will require additional experiments, analyses and clarifications to be fully addressed.

1.Specifically, in the competition context group, the dominant and subordinate mice are accessing varied volumes of reward compared to the Together and Alone contexts in which the number of trials are kept constant. Therefore, one concern is that different volumes of rewards accessed over time could contribute to the differential levels of activity observed across social contexts. This would be a particular concern for subordinate mice that are only getting a small subset of the rewards. One way to address this would be to yoke the number of trials across conditions (specifically in the Together and Alone conditions).

2.It is unclear if in the together group, the trials are occurring simultaneously or are they offset. This should be clarified in the methods.

3.It is unclear how brain regions are defined? Are they mapped onto an atlas ? Are you counting all cells in a region ? Or is it kept to a predefined area per region ?

4.The sensitivity measure, K, in the Elo score selected to be 20. How was this determined?

5.Are the territory-based and reward-based ranks stable across time ? This would be really important to determining how results on the final day correlate with what might be happening during social competition. Also a supplementary figure showing correlations between both would be extremely helpful in this regard. Additionally, are social hierarchies as determined by both of these methods transitive ?

6.It is an interesting idea by the authors to use port entry as a metric of motivation, but it is also possible that port entry here is a measure of impulsivity. Given the absence of null port, the authors cannot really distinguish between these possibilities.

7.In several figures, the authors use an one-way ANOVA to determine statistical significance (e.g Fig 2A, B etc). However, the right stat is to run a 2-way ANOVA with region and context as factors.

8.If two regions show high levels of c-FOS activity, aren't they more likely to be correlated? E.g ACC vs PL in the Together context. What does the shuffled correlation look like, if the labels are shuffled while keeping number of cells active constant ? Would be important to look at what the null-distribution looks like. In general, all correlation plots could use shuffled comparisons as controls.

9.Calculating numerous correlations increases the risk of a type I error, i.e., to erroneously conclude the presence of a significant correlation. To avoid this, the level of statistical significance of correlation coefficients should be adjusted. If the authors are underpowered, the limitation should be addressed in the discussion.

10.The authors show CA1 activity differentially correlates with competition and territorial dominance, it is not clear why this would be expected and it is not explored by the authors.

11.In fig 2-1, why are there only 6 data points when there are 10 animals in the competition context according to the methods section ?

12. Also, does correlation in the competition context look different if the animals are grouped as winners/ losers of the final competition bout.

13. Instead of compressed rank, does use of the actual Elo-scores change correlations (since the ranks compress the dynamic range).

Reviewer 2:

Dear Editor,

Thank you for the opportunity to review the manuscript entitled as "Prefrontal and subcortical c-Fos mapping of reward responses across competitive and social contexts." This manuscript investigates neuronal activation patterns, using c-Fos immunohistochemistry, in response to reward under different social scenarios: 'alone', '[direct] competition', and 'together' (witnessed competition). The study aims to explore the neural correlates of competitive behavior, particularly in relation to social dominance within this rewarded context. While the central research question is highly relevant and the comparative approach across three distinct paradigms is potentially interesting, the study in its current form is preliminary and suffers from significant methodological limitations, insufficient data analysis and presentation, and a lack of crucial controls. These issues prevent the findings from being robustly interpreted and significantly limit the manuscript's contribution.

Major Concerns:

1. Preliminary nature and scope: While the comparison between the 'alone', '[direct] competition', and 'together (witnessed competition)' conditions is a potentially valuable aspect of the study design, the overall investigation feels preliminary. It provides only an initial, rough map of potential neural correlates without sufficient depth or validation to draw firm conclusions. It represents perhaps the first step of a much larger required investigation.

2. Inadequate assessment of social hierarchy: The study aims to link social dominance to neural activation during competition for a reward. However, other than 'competition context', social dominance is assessed solely through territorial urine marking. While urine marking can be an indicator, it represents only one facet of social status and may not fully capture the complexities of dominance relevant to social hierarchy in group-housed animals. To strengthen this aspect, dominance should be evaluated using multiple established behavioral assays (e.g., tube test, warm spot test, agonistic encounters). This multi-dimensional assessment would allow for a more nuanced understanding of how different aspects of social rank relate to competitive behavior and associated neural activity.

3. Lack of critical behavioral detail on aggressive behavior: A significant omission is the lack of information regarding aggressive interactions during the 'competition' paradigm. Aggression is a potent modulator of social behavior and neural activity, including c-Fos expression in relevant circuits. Without quantifying or reporting the frequency and nature of any aggressive encounters between competitors, it is impossible to disentangle neural activity specifically related to resource competition from that associated with agonistic behavior.

4. Limitations of c-Fos mapping and need for validation: The study relies exclusively on c-Fos immunohistochemistry as a proxy for neuronal activity. While c-Fos is a widely used marker for identifying recently active neurons, its expression is correlational and does not establish causality as was briefly mentioned in the discussion (page 9). Findings derived solely from c-Fos mapping require further histological and functional validation to demonstrate that the activity in the identified brain regions is necessary or sufficient for the observed behaviors. The manuscript would need to incorporate, some cell-type characterization to explore the circuitry and to functionally probe the role of the identified circuits in mediating competitive/social behavior through excitation/inhibition via chemogenetics, optogenetics, or pharmacological interventions).

5. Inadequate data presentation and visualization: The current presentation of the results hinders a clear understanding of the findings. The reliance primarily on many correlation graphs makes it difficult for the reader to synthesize the information and discern meaningful patterns across brain regions, behavioral measures, and experimental conditions. The visuals feel preliminary and lack integration. More sophisticated data visualization methods are strongly suggested. For example, exploring cluster analysis might reveal underlying patterns in neural activation profiles across conditions or individuals. A clearer, more integrated, and insightful visual representation of the data is essential.

In summary, while the manuscript addresses an interesting topic in social neuroscience, it is currently too preliminary and suffers from substantial methodological and analytical weaknesses. Addressing these fundamental issues would require significant additional experimentation, analysis, and potentially a reframing of the study. As it stands, the manuscript does not provide a sufficiently rigorous or conclusive contribution to warrant publication.

## Author Response

Reviewer 1:

In this article, authors use c-FOS labeling to systematically map how reward responses change across social contexts. They find intriguing differences in which subregions are functionally correlated depending on social context and social rank. Intriguingly, they find significant differences in how activity in several regions in the social decision-making network correlate with reward-based rank vs territory-based ranking of social hierarchy. Additionally, they find no correlation between social ranks generated by these two methods. This is a particularly interesting finding that informs our understanding of social group dynamics and the validity of these different social dominance assays. The authors should consider expanding on this theme in the discussion. While the paper has several interesting findings, there are some significant concerns regarding the methodological details and the interpretations of findings will require additional experiments, analyses and clarifications to be fully addressed.

1. Specifically, in the competition context group, the dominant and subordinate mice are accessing varied volumes of reward compared to the Together and Alone contexts in which the number of trials are kept constant. Therefore, one concern is that different volumes of rewards accessed over time could contribute to the differential levels of activity observed across social contexts. This would be a particular concern for subordinate mice that are only getting a small subset of the rewards. One way to address this would be to yoke the number of trials across conditions (specifically in the Together and Alone conditions).

We agree with the reviewer that differences in reward volume could influence c-Fos expression. Unfortunately, yoking across the current subject data is impossible. However, in the Together and Alone context, all mice consumed the same number of rewards (19 rewards specifically). To address the possibility that number of rewards impacts c-Fos expression, we conducted correlations between the number of rewards obtained and c-Fos+ cell counts across all brain regions during the Competition context. These analyses revealed no significant correlations after multiple comparison corrections (see Results section and Fig. 2-2), suggesting that reward volume alone does not account for the observed neural differences. The only region that had a trend of correlation between number of rewards obtained in the competition was ACC, and we discussed this possibility in the discussion of the article.

2. It is unclear if in the together group, the trials are occurring simultaneously or are they offset. This should be clarified in the methods.

We thank the reviewer for bringing up this oversight. We have clarified this methodological detail under the section titled "EXPERIMENTS: GROUPS AND PERFUSIONS" of the methods on page 3 "When a tone played within the operant chamber, rewards were dispensed simultaneously to both mice, with each retrieving the same number of rewards, allowing both animals to experience the same number of trials with simultaneous exposure to tone and reward." 3. It is unclear how brain regions are defined? Are they mapped onto an atlas ? Are you counting all cells in a region ? Or is it kept to a predefined area per region ? We thank the reviewer for pointing out this incomplete information in the methods of our original submission. We have made several changes to address this concern:

In the methods section on page 4, we add the following language: "Within each section, a fixed field of view corresponding to the microscope's 20× magnification window was used to consistently sample a representative area of the region. ROIs were manually delineated within this field of view, and the same imaging parameters were applied across animals and brain regions (Fig. 2-1)".

We have also added a new supplemental figure explaining our region of interest for cell counting (copied below).

Figure 2-1. Example regions of interest for each brain region. (A-H) Average size and shape of the region of interest (ROI) for a given brain area. (A-G): 4.4 x 104 µm2 square (ACC, PL, IL, NAc, LH, BLA, MD). (H) 2.0 x 104 µm2 of pyramidal layer of (vCA1).

4. The sensitivity measure, K, in the Elo score selected to be 20. How was this determined? We selected the Elo rating method to quantify social dominance because it accommodates the temporal dynamics of interaction and allows dominance estimates to evolve across time, which is a critical feature for modeling social hierarchies in behavioral experiments (Neumann et al., 2011, Animal Behaviour). Among commonly used ranking methods, Elo-rating and David's score correlate and outperform other approaches such as ADAGIO and I&SI in terms of accurately modeling dominance relationships (Neumann at al., 2018, Plos One).

While Elo-rating requires specification of a constant k value to determine the magnitude of rating updates after each interaction, there is no universally accepted standard for this parameter. Rather, k is typically chosen within an arbitrary range, commonly reported to be between 16 and 200 depending on the structure of the data and the desired sensitivity of the model (Neumann et al., 2011, Animal Behaviour; Neumann et al., 2023, Methods in Ecology and Evolution). Newton-Fisher (2017) demonstrated that varying k values can be used to reflect different intensities of aggressive interactions and noted that, although k impacts how rapidly ratings change in the short term, it has minimal long-term influence on the final dominance ranks. Based on Albers and De Vries (2000), large K values result in a bias towards early contests. Given that our reward competitions and urine marking assays are done in a randomized round-robin manner, we do not want to give extra weight to early contests and thus use the lower k value of 20 used in by Albers and De Vries. Importantly, Albers and De Vries (2000) showed that the resulting ranks correlated with another dominance ranking method, whether you used k=20 or k=200.

Given these considerations, we adopted a conservative value of k=20 for our study. This choice limits volatility in rating updates, ensuring that transient behavioral fluctuations do not overly influence the stability of inferred rank. However, we now have verified that our results are consistent if we vary the k from k=10 to k=40, showing the same final ranks in 178 out of 192 measurements (see figures below; columns contain different k values and each row is an individual cage). By prioritizing robustness over responsiveness, this parameterization reflects our goal of capturing stable dominance patterns while remaining grounded in prior methodological precedent.

We have updated our methods to include the following additional information on how we selected our k parameter: "Given that the reward competitions and urine marking assays are done in a randomized round-robin manner, as to not give extra weight to early contests, k = 20 was used (Albers and De Vries, 2001)." 5. Are the territory-based and reward-based ranks stable across time? This would be really important to determining how results on the final day correlate with what might be happening during social competition. Also a supplementary figure showing correlations between both would be extremely helpful in this regard. Additionally, are social hierarchies as determined by both of these methods transitive? In our study, we only conducted one or two matches (sessions) per pair per test type, therefore we cannot assess stability across time. All Elo scores were calculated based on behavior observed during that period, and all animals remained in stable home cages throughout the experiment. Thus, the ranks used in our analyses reflect each animal's social status within a consistent timeframe capturing the current rank at the time of c-Fos staining.

Due to our limited number of mice within a hierarchy (4 mice per cage), it is adequate to calculate a Directional Consistency Index (DCI) per cage across the assays to assess transitivity. Urine marking assays showed higher DCI values, indicating greater directional consistency across ranks, than reward competition DCI values. However, the reward competition and urine marking assay have different numbers of trials and the values are consistent with those published in prior literature (Cum et al 2024, eNeuro).

6. It is an interesting idea by the authors to use port entry as a metric of motivation, but it is also possible that port entry here is a measure of impulsivity. Given the absence of null port, the authors cannot really distinguish between these possibilities.

We acknowledge the reviewer's important point. Since we cannot distinguish between motivation and impulsivity, we have reworded this section to use the more general term 'reward-seeking behavior.' 7. In several figures, the authors use an one-way ANOVA to determine statistical significance (e.g Fig 2A, B etc). However, the right stat is to run a 2-way ANOVA with region and context as factors.

Originally, our intention was not to compare brain regions, which is why we opted for the one-way ANOVA design focused on our variable of interest: context. However, we acknowledge that to provide the most comprehensive description of the data, a 2-way ANOVA offers readers the opportunity to understand relative activation across regions. Given that the area counted was not the same for all regions (see new Figure 2-1), we now report Figure 2 in density of c-Fos to be able to perform a cross-regional comparison. As we have multiple measurements per subject across multiple brain regions, we opted for a mixed effects ANOVA that would account for repeated measures. The methods now state: "For Figure 2, Type III two-way linear mixed-effects ANOVA using Satterthwaite's method were conducted. First a model was conducted on cell densities with brain region (ACC, PL, IL, NAc, MD, BLA, LH, vCA1) and context (Competition, Together, Alone) as fixed effects and mouse ID as random effects. A second model was conducted on the percent change in c-Fos+ cell density from the Alone baseline, with context (Competition, Together) and brain region (ACC, PL, IL, NAc, MD, BLA, LH, vCA1) as fixed effects and mouse ID as a random effect." 8. If two regions show high levels of c-FOS activity, aren't they more likely to be correlated? E.g ACC vs PL in the Together context. What does the shuffled correlation look like, if the labels are shuffled while keeping number of cells active constant ? Would be important to look at what the null-distribution looks like. In general, all correlation plots could use shuffled comparisons as controls.

To address the concern that high c-Fos activity in two regions might lead to spurious correlations, we performed a permutation test in which we shuffled animal labels within each brain region per condition. This approach generated a null distribution for each correlation while preserving the average c-Fos activation levels within a region and enabled us to determine whether the observed correlations are stronger than expected by chance. We calculated both the percentile at which the observed correlation coefficient was found and took the p-value derived from the permutation test, adjusting it for multiple comparisons (discussed further below). We now include these results in Tables 3-5. We did not find any effects of average c-Fos level on the strength of the correlation coefficient. Due to the limitation of our small sample size and to avoid type II errors, we highlight correlations with coefficients above the 97.5th or below the 2.5th percentile.

The following has also been added to the methods section under STATISTICAL ANALYSIS: "To analyze functional relationships between brain regions, we used a permutation test (scipy.stats.permutation_test). For each correlation, we generated a null distribution by randomly shuffling cell densities within regions across 2,000 permutations while calculating Pearson's correlation coefficients. For each permutation subject labels were shuffled within a given brain region, thus maintaining the mean c-Fos values per region. The Benjamini-Hochberg False Discovery Rate procedure was applied to two tailed p-values derived from the permutation test. For cross-regional correlations, FDR adjustments were applied to all brain region pairs within each experimental context separately (Competition, Alone, Together). These values are shown in Tables 3-5. The same permutation procedure was applied to all correlational analyses between dominance measures (relative rank and Elo score) and c-Fos cell counts per brain region. For rank correlations, FDR adjustments were applied within each unique combination of rank metric, assay, and experimental context, correcting across all 8 brain regions simultaneously e.g., all 8 regional correlations for relative rank derived from the urine marking assay within the Competition context were adjusted together." 9. Calculating numerous correlations increases the risk of a type I error, i.e., to erroneously conclude the presence of a significant correlation. To avoid this, the level of statistical significance of correlation coefficients should be adjusted. If the authors are underpowered, the limitation should be addressed in the discussion.

The reviewer makes an important point. To remedy this problem, we applied FDR (Benjamini-Hochberg) corrections across all correlation analyses in the manuscript. After this adjustment, several cross-regional c-Fos correlations, particularly those in the Together and Competition conditions, no longer reached statistical significance. We adjusted the results and discussion sessions accordingly. In contrast, many significant cross-regional correlations in the Alone context remained present, and thus our main conclusion remained valid. Several significant correlations were reduced for social rank correlations as well. Given the exploratory nature of our study, we highlight observations with correlation coefficients that are above the 97.5th or below the 2.5th percentile as stated above. We do not claim statistical significance for these observations and this limitation is now explicitly stated in the Discussion section.

10. The authors show CA1 activity differentially correlates with competition and territorial dominance, it is not clear why this would be expected and it is not explored by the authors.

We thank the reviewer for pointing out that we did not explore this finding. We now added the following to our discussion: "Further context-dependent neural activity was observed in the hippocampus. We found that vCA1 activity correlated with reward-based rank in the Alone context and with agonistic-based rank in the competition context. These preliminary findings suggest that vCA1 may integrate social and motivational cues in a context-specific manner. The observed pattern of vCA1 activity in the Alone context, in contrast to its pattern to agonistic rank in a competitive context, highlights vCA1 potential sensitivity to the context. In the Alone context, the reward remains the most salient stimulus while in the Competition context, the competitor must also be accounted for. This aligns with recent findings that vCA1 neurons encode the conjunction of social stimuli, their spatial location, and environmental context (Wen-Yi Wu et al., 2024), supporting the idea that vCA1 plays a key role in integrating social experience with contextual cues." 11. In fig 2-1, why are there only 6 data points when there are 10 animals in the competition context according to the methods section? We appreciate the reviewer pointing out this point. Due to camera failure, two videos from the round-robin competition were lost. This directly impacted the sample size for calculating the number of rewards obtained during the competition context (Figure 2-2). Since each video contained reward data for two mice, and two videos were lost we went from 10 subjects - (2 mice x 2 videos) = 6 remaining subjects. This information has been added to the methods. This is also highlighted in the methods section under Cagemate Ranks, which now reads: "Due to camera failure, several round-robin competition videos were lost, directly impacting the total sample for the calculations of the number of rewards obtained during the competition context and mean number of c-Fos+ cells across brain regions (Figure 2-2)." 12. Also, does correlation in the competition context look different if the animals are grouped as winners/ losers of the final competition bout.

Given the limited number of animals with videos in the final competition bout, separating the data per winner and loser is not feasible. However, we did perform a correlation of number of wins with c-Fos cell counts that is shown in Figure 2-2. This analysis suggests that in most brain regions, total number of wins has little impact on overall c-Fos expression with the exception of ACC.

13. Instead of compressed rank, does use of the actual Elo-scores change correlations (since the ranks compress the dynamic range).

We used relative rank to normalize ranks across cages because not every mouse from each cage was included in a given group, and mice only competed against their cagemates. Since subjects from each cage were randomized across conditions (Competition, Together, Alone), the same Elo score found in different cages could reflect different relative ranks. We have added language to the methods to better justify and explain our selection. "We opted to use a discrete rank system (1 to 4) rather than continuous Elo scores because the mice used in each testing group were selected from different home cages, and not all mice within each home cage were included in the experiment, thus the same Elo score value across cages could indicate different relative ranks." Below we show what correlations between Elo score and c-Fos for the reviewer to see. However, for simplicity we do not include these scatterplots in our manuscript. Instead, we reported how consistent the c-Fos and rank correlations are when using Elo score vs relative rank in summary figures for each social rank (Fig 4D, 5D, 6D, 7F, 8F and 9F).

Reviewer 2:

Dear Editor, Thank you for the opportunity to review the manuscript entitled as "Prefrontal and subcortical c-Fos mapping of reward responses across competitive and social contexts." This manuscript investigates neuronal activation patterns, using c-Fos immunohistochemistry, in response to reward under different social scenarios: 'alone', '[direct] competition', and 'together' (witnessed competition). The study aims to explore the neural correlates of competitive behavior, particularly in relation to social dominance within this rewarded context. While the central research question is highly relevant and the comparative approach across three distinct paradigms is potentially interesting, the study in its current form is preliminary and suffers from significant methodological limitations, insufficient data analysis and presentation, and a lack of crucial controls. These issues prevent the findings from being robustly interpreted and significantly limit the manuscript's contribution.

Major Concerns:

1. Preliminary nature and scope: While the comparison between the 'alone', '[direct] competition', and 'together (witnessed competition)' conditions is a potentially valuable aspect of the study design, the overall investigation feels preliminary. It provides only an initial, rough map of potential neural correlates without sufficient depth or validation to draw firm conclusions. It represents perhaps the first step of a much larger required investigation.

We appreciate the reviewer's perspective. We acknowledge indeed that this study is a first step towards future research focused on mechanisms. To our knowledge, this is the first study to systematically compare activity levels across distinct reward and social contexts while examining multiple dominance metrics to understand how brain activity relates to social dominance. The correlation patterns we observe provide concrete hypotheses for future mechanistic research.

2. Inadequate assessment of social hierarchy: The study aims to link social dominance to neural activation during competition for a reward. However, other than 'competition context', social dominance is assessed solely through territorial urine marking. While urine marking can be an indicator, it represents only one facet of social status and may not fully capture the complexities of dominance relevant to social hierarchy in group-housed animals. To strengthen this aspect, dominance should be evaluated using multiple established behavioral assays (e.g., tube test, warm spot test, agonistic encounters). This multi-dimensional assessment would allow for a more nuanced understanding of how different aspects of social rank relate to competitive behavior and associated neural activity.

Our lab has published work about the multi-faceted dimensionality of social dominance, and we could not agree with the reviewer more wholeheartedly. We appreciate the reviewer's comment and have added agonistic behaviors to our repertoire of social dominance metrics. Thus, the manuscript now includes 3 dominance measures. Most studies of social hierarchy only report one dominance measurement; therefore, our study goes above the standards of the field by reporting 3 distinct metrics.

3. Lack of critical behavioral detail on aggressive behavior: A significant omission is the lack of information regarding aggressive interactions during the 'competition' paradigm. Aggression is a potent modulator of social behavior and neural activity, including c-Fos expression in relevant circuits. Without quantifying or reporting the frequency and nature of any aggressive encounters between competitors, it is impossible to disentangle neural activity specifically related to resource competition from that associated with agonistic behavior Indeed, aggression does impact brain activity. To address this concern, we performed an additional behavioral analysis of agonistic interactions, quantifying the total number of aggressive behaviors (bites, chases, and fights) initiated by each mouse during the reward competition sessions. We incorporated this agonistic rank into our manuscript. Figures 6 and 7 are centered on showing how agonistic ranks correlate with c-Fos activity in our regions of interest. We found a significant correlation between agonistic dominance and activity in the MD in the Together context (r = 0.8359, p = 0.0160) and, although not significant after multiple comparison correction, activity in vCA1 in the Competitive context had a correlation coefficient above the 97.5th percentile of a null distribution of coefficients (r = 0.8359, p = 0.5517). Even though the reward-based and agonistic-based rankings had trending correlations between each other, their relationships to c-Fos activity differed. This suggests that although these facets of dominance may influence the resulting rank of an individual similarly, their effect on neural activity depends on the reward context. We believe that the inclusion of this agonistic rank strengthens our study.

4. Limitations of c-Fos mapping and need for validation: The study relies exclusively on c-Fos immunohistochemistry as a proxy for neuronal activity. While c-Fos is a widely used marker for identifying recently active neurons, its expression is correlational and does not establish causality as was briefly mentioned in the discussion (page 9). Findings derived solely from c-Fos mapping require further histological and functional validation to demonstrate that the activity in the identified brain regions is necessary or sufficient for the observed behaviors. The manuscript would need to incorporate, some cell-type characterization to explore the circuitry and to functionally probe the role of the identified circuits in mediating competitive/social behavior through excitation/inhibition via chemogenetics, optogenetics, or pharmacological interventions).

We appreciate and agree with the reviewer's understanding and agree that correlations do not imply causation. We have ensured that we made no such claims in the manuscript, and as the reviewer mentioned, we do acknowledge the limitations of c-Fos studies in our discussion section. We believe correlation studies, especially exploratory studies such as ours, are valuable for the field and merit publication.

5. Inadequate data presentation and visualization: The current presentation of the results hinders a clear understanding of the findings. The reliance primarily on many correlation graphs makes it difficult for the reader to synthesize the information and discern meaningful patterns across brain regions, behavioral measures, and experimental conditions. The visuals feel preliminary and lack integration. More sophisticated data visualization methods are strongly suggested. For example, exploring cluster analysis might reveal underlying patterns in neural activation profiles across conditions or individuals. A clearer, more integrated, and insightful visual representation of the data is essential.

We thank the reviewer for this wonderful suggestion. We have added some integrated visuals for both region-to-region correlations (Figure 3D), and c-Fos and rank correlations (Figures 4-9). We think these visualizations enhance our manuscript and allow the readers to synthesize the findings in a holistic way.
