# Supplementary figures and images for: Prefrontal and Subcortical c-Fos Mapping of Reward Responses across Competitive and Social Contexts
Source: eNeuro. 2025 Nov 6;12(11):ENEURO.0158-25.2025. doi: 10.1523/ENEURO.0158-25.2025 (PMC12611407; doi:10.1523/ENEURO.0158-25.2025)

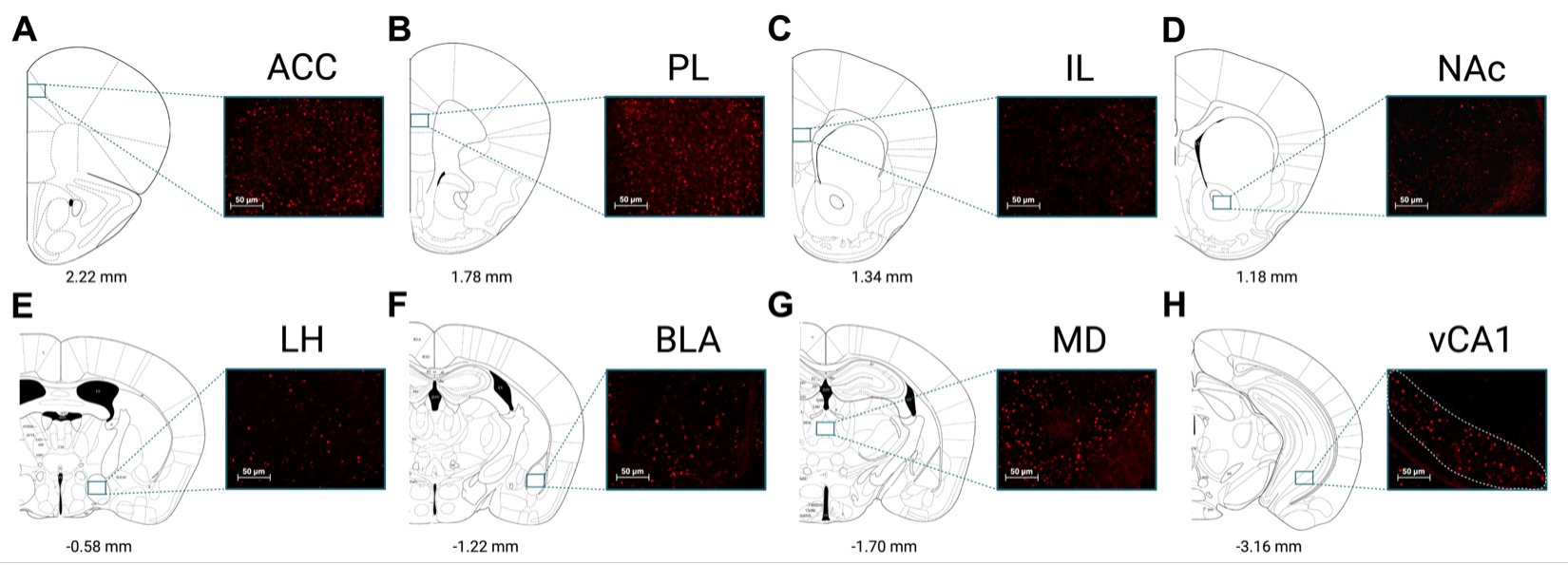

Supplement: Figure 2-1 — Example regions of interest for each brain region. (A-H) Average size and shape of the region of interest (ROI) for a given brain area. (A-G): 4.4 × 10^4^ µm2 square (ACC, PL, IL, NAc, LH, BLA, MD). (H) 2.0 × 10^4^ µm2 of pyramidal layer of (vCA1). Download Figure 2-1, TIF file. [file eneuro-12-ENEURO.0158-25.2025-s002.tif]

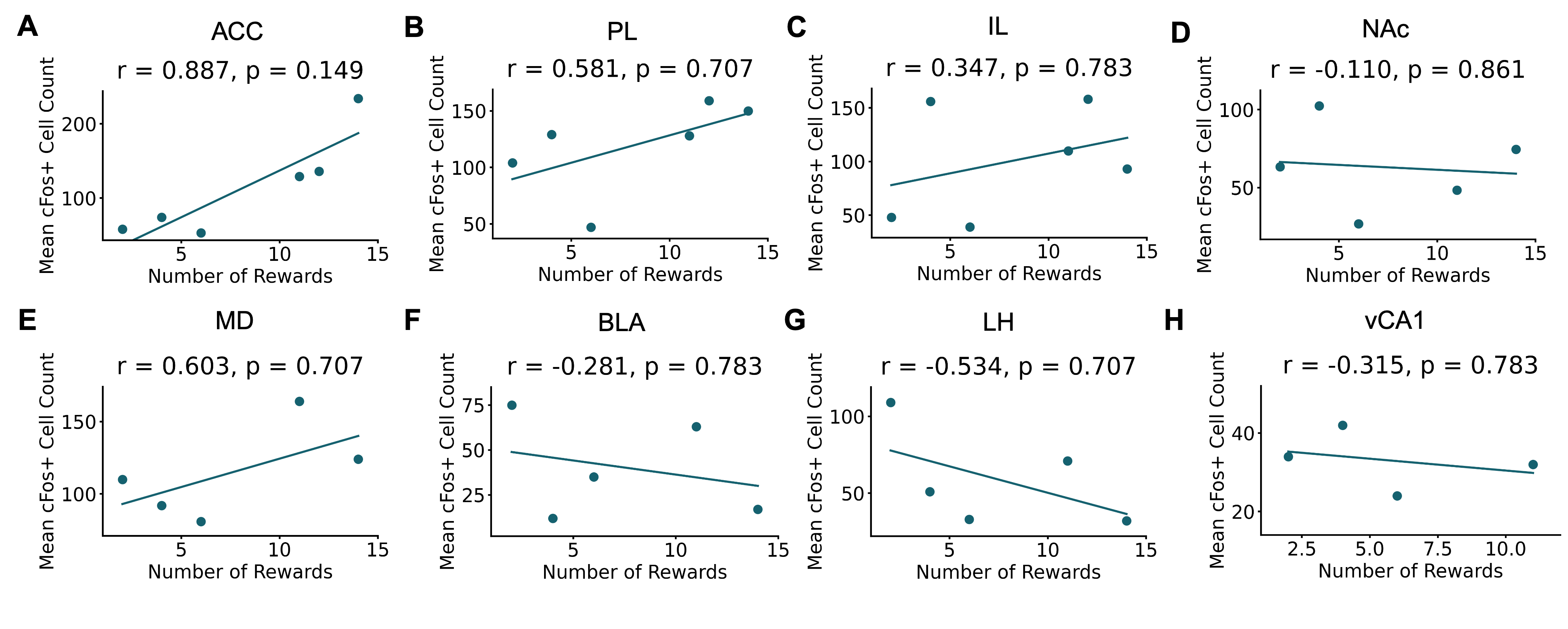

Supplement: Figure 2-2 — Rewards won during Competition context. (A-H) Scatterplots for the number of rewards obtained during the competition context and mean number of c-Fos + cells across brain regions. Each dot represents an individual animal, with lines of best-fit derived from a linear regression analysis. P-values were adjusted using a Benjamini-Hochberg False Discovery Rate procedure. Pearson correlation coefficient (r) and associated adjusted p-values are displayed above each plot. Download Figure 2-2, TIF file. [file eneuro-12-ENEURO.0158-25.2025-s003.tif]

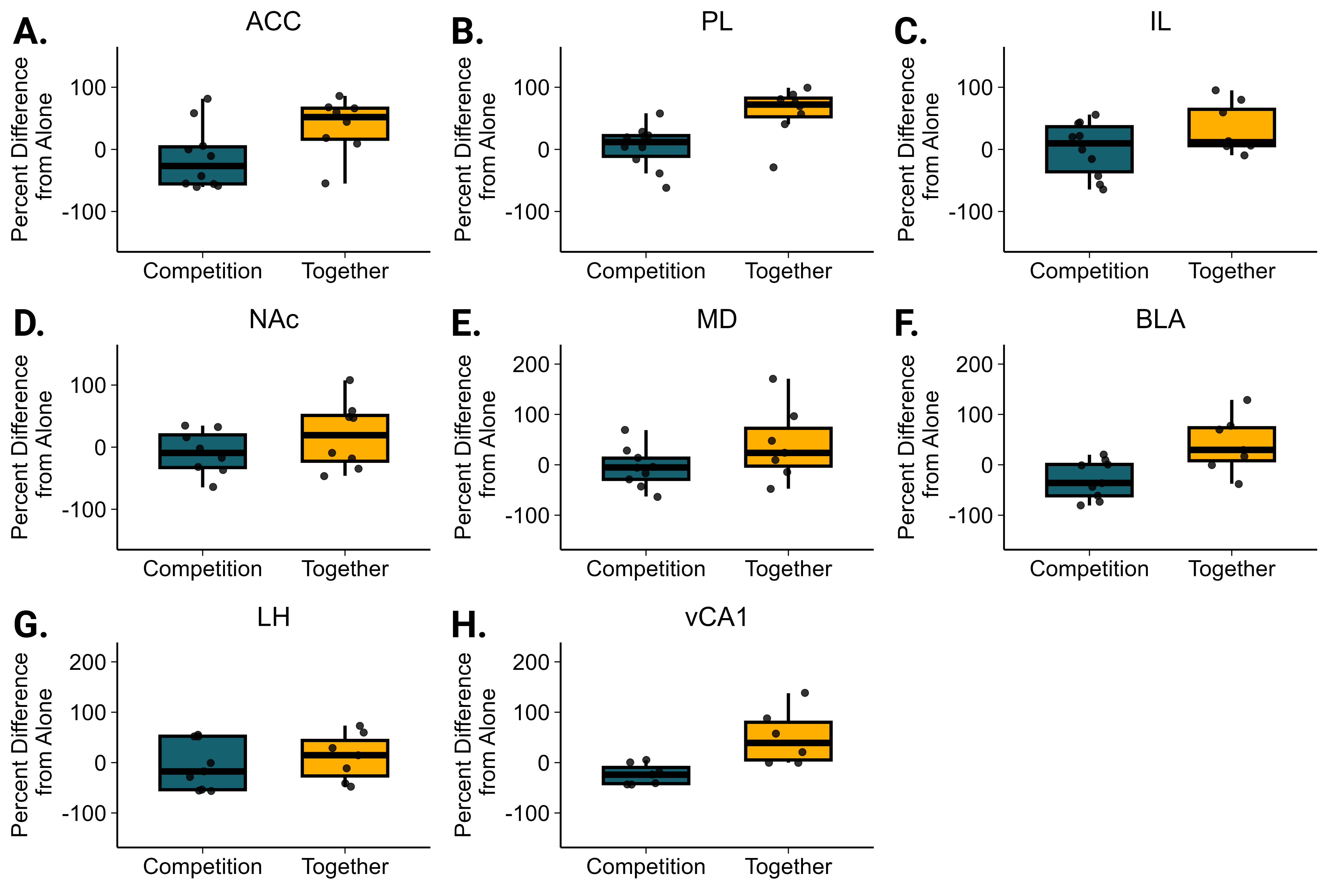

Supplement: Figure 2-3 — Individual brain regions c-Fos densities normalized to Alone baseline. (A-H) Percent differences in c-Fos⁺ cell density calculated from Alone baseline across conditions for each brain region. Each data point represents the difference from baseline for an individual subject for that brain region. Positive values indicate increased activity relative to Alone; negative values indicate decreased activity. Box plots show median and interquartile ranges. Download Figure 2-3, TIF file. [file eneuro-12-ENEURO.0158-25.2025-s004.tif]
